# *Clostridioides difficile* toxin B alone and with pro-inflammatory cytokines induces apoptosis in enteric glial cells by activating three different signalling pathways mediated by caspases, calpains and cathepsin B

**DOI:** 10.1007/s00018-022-04459-z

**Published:** 2022-07-22

**Authors:** Katia Fettucciari, Flavien Marguerie, Alessandro Fruganti, Andrea Marchegiani, Andrea Spaterna, Stefano Brancorsini, Pierfrancesco Marconi, Gabrio Bassotti

**Affiliations:** 1 Biosciences and Medical Embryology Section, Department of Medicine and Surgery, Medical School, University of Perugia, Edificio B-IV piano, Piazza Lucio Severi 1, 06132 Perugia, Italy; 2 School of Biosciences and Veterinary Medicine, University of Camerino, Via Circonvallazione 93/95, 62024 Matelica (MC), Italy; 3 General Pathology Section, Department of Medicine and Surgery, University of Perugia, Via Mazzieri 3, 05100, Terni - Piazza Lucio Severi 1, 06132 Perugia, Italy; 4 Gastroenterology, Hepatology and Digestive Endoscopy Section, Department of Medicine and Surgery, Medical School, University of Perugia, Piazza Lucio Severi 1, 06132 Perugia, Italy; 5 Gastroenterology and Hepatology Unit, Santa Maria Della Misericordia Hospital, Piazzale Menghini 1, 06156 Perugia, Italy

**Keywords:** *Clostridioides difficile*, *C. difficile* toxin B (TcdB), Enteric glial cells (EGCs), Apoptosis, Cell death, Cell cycle, Calpains, Caspases, Cathepsin B, Tumour necrosis factor alpha (TNF-α), Interferon gamma (IFN-γ), Cysteine proteases

## Abstract

*Clostridioides difficile* infection (CDI) causes nosocomial/antibiotic-associated gastrointestinal diseases with dramatically increasing global incidence and mortality rates. The main *C. difficile* virulence factors, toxins A and B (TcdA/TcdB), cause cytopathic/cytotoxic effects and inflammation. We demonstrated that TcdB induces caspase-dependent, mitochondria-independent enteric glial cell (EGC) apoptosis that is enhanced by the pro-inflammatory cytokines TNF-α and IFN-γ (CKs) by increasing caspase-3/7/9 and PARP activation. Because this cytotoxic synergism is important for CDI pathogenesis, we investigated the apoptotic pathways involved in TcdB- and TcdB + CK-induced apoptosis indepth. EGCs were pre-treated with the inhibitors BAF or Q-VD-OPh (pan-caspase), Z-DEVD-fmk (caspase-3/7), Z-IETD-fmk (caspase-8), PD150606 (calpains), and CA-074Me (cathepsin B) 1 h before TcdB exposure, while CKs were given 1.5 h after TcdB exposure, and assays were performed at 24 h. TcdB and TcdB + CKs induced apoptosis through three signalling pathways activated by calpains, caspases and cathepsins, which all are involved both in induction and execution apoptotic signalling under both conditions but to different degrees in TcdB and TcdB + CKs especially as regards to signal transduction mediated by these proteases towards downstream effects (apoptosis). Calpain activation by Ca^2+^ influx is the first pro-apoptotic event in TcdB- and TcdB + CK-induced EGC apoptosis and causes caspase-3, caspase-7 and PARP activation. PARP is also directly activated by calpains which are responsible of about 75% of apoptosis in TcdB and 62% in TcdB + CK which is both effector caspase-dependent and -independent. Initiator caspase-8 activation mediated by TcdB contributes to caspase-3/caspase-7 and PARP activation and is responsible of about 28% of apoptosis in both conditions. Caspase-3/caspase-7 activation is weakly responsible of apoptosis, indeed we found that it mediates 27% of apoptosis only in TcdB. Cathepsin B contributes to triggering pro-apoptotic signal and is responsible in both conditions of about 35% of apoptosis by a caspase-independent manner, and seems to regulate the caspase-3 and caspase-7 cleaved fragment levels, highlighting the complex interaction between these cysteine protease families activated during TcdB-induced apoptosis. Further a relevant difference between TcdB- and TcdB + CK-induced apoptosis is that TcdB-induced apoptosis increased slowly reaching at 72 h the value of 18.7%, while TcdB + CK-induced apoptosis increased strongly reaching at 72 h the value of 60.6%. Apoptotic signalling activation by TcdB + CKs is enriched by TNF-α-induced NF-κB signalling, inhibition of JNK activation and activation of AKT. In conclusion, the ability of *C. difficile* to activate three apoptotic pathways represents an important strategy to overcome resistance against its cytotoxic activity.

**Supplementary Information:**

The online version contains supplementary material available at 10.1007/s00018-022-04459-z.

## Introduction

*Clostridioides difficile* (*C. difficile)* infection (CDI) causes nosocomial/antibiotic-associated diarrhoea and pseudomembranous colitis, and its global incidence and mortality have dramatically increased [1–3]. The major virulence factors of *C. difficile* are toxins A (TcdA) and B (TcdB), although TcdB is more potent (~ 1000-fold) than TcdA [4–8]; both cause glucosylation of Rho GTPases, cytopathic/cytotoxic effects and inflammation [4–8].

Enteric cells, such as enterocytes, colonocytes and enteric neurons, are highly susceptible to the cytotoxic effects of TcdA and TcdB. Many studies concerning the pathogenesis of *C. difficile* have been conducted using these cell types, immune cells or cell lines of colorectal carcinomas and of the pancreas [4–8]. Recently, Fettucciari et al. showed that TcdB also affects enteric glial cells (EGCs), causing early cytopathic effects (cell rounding, Rac1 glucosylation, cell cycle arrest) and apoptosis, in a caspase-dependent but mitochondria-independent manner [9]. TcdB-induced EGC apoptosis is essentially mediated by the activation of caspase-3 and poly(adenosine diphosphate-ribose) polymerase (PARP) and by the delayed activation of caspase-7 without alteration of the expression of pro- and anti-apoptotic B-cell lymphoma 2 (Bcl-2) family members [9, 10].

During CDI, cells of the gut wall, including EGCs, are subjected to strong stimulation by pro-inflammatory cytokines such as interleukin-1 beta (IL-1β), tumour necrosis factor alpha (TNF-α) and interferon gamma (IFN-γ) [4–8, 11–14]. Some studies of the effects of these pro-inflammatory cytokines on EGCs found that EGCs are highly resistant to apoptosis following treatment with pro-inflammatory cytokines such as IFN-γ, TNF-α, IL1-β, and interleukin-6 (IL-6) alone or in combination with IL-1β and IL-6 [3, 11–14]. However, Fettucciari et al. have shown that EGCs become more susceptible to apoptosis induced by TcdB when they are stimulated with the combination of the TNF-α plus IFN-γ (CKs) before, during or after treatment with TcdB [9]. This increase in apoptosis was found to be correlated with the increased activation of caspase-3, caspase-7, and caspase-9 and PARP without a change in the expression of Bcl-2 family members [9]. However, the molecular mechanisms responsible for these effects were not defined. This synergistic effect could be very important for the pathogenesis of CDI since primary CDI and relapses might be favoured by a mechanism that involves enhancement of the toxicity of TcdB against EGCs by CKs and their network in an environment characterized by strong dysmicrobism [15]. Moreover, when TcdB enters the systemic circulation of patients affected by severe CDI, its toxic activity against various organs (extraintestinal manifestations) can be strongly enhanced due to the presence of a systemic cytokine storm, in which the concentrations of pro-inflammatory cytokines such as TNF-α and IFN-γ reach significant levels in the circulation [16].

Analysis of the mechanisms of apoptosis induced by TcdA [17–23] or TcdB [24–29] in several different cell types demonstrated that the executioner caspase-3 plays a key role in TcdA- and TcdB-induced apoptosis [4–8]. Further, activation of the executioners caspase-6 and caspase-7 was also described [17, 20]. The executioner caspases-3, caspase-7 and caspase-6 are activated by two different initiator caspases, caspase-8 and caspase-9 [30, 31]. Caspase-8 is a major player in the extrinsic apoptotic pathway that can be activated via trans-membrane death receptors such as tumour necrosis factor receptor 1, IFN-γ, Fas, or TNF-related apoptosis-inducing ligand receptors; caspase-8, once activated, directly cleaves/activates the effector caspases caspase-3 and caspase-7 or cleaves/activates the BH3-interacting domain death agonist (Bid) protein, leading to activation of the intrinsic apoptotic pathway, which results in effector caspase activation [30–33]. However, there is evidence showing that the initiator caspases caspase-8 and caspase-9 can also act as executioner caspases [30–35]. Almost all caspases are known to modify PARP [30–33, 35]; it has been reported that caspase-8 can relocate in the nucleus, where it may cleave PARP which mediates the execution phase of apoptosis without involvement of effector caspases [34]. Caspase-8 also plays a role in TcdA-induced apoptosis [17, 20]. Caspase-9, representing the intrinsic pathway, is activated by the apoptosome containing cytochrome c released from mitochondria [30, 31]. Intrinsic pathway activation by *C. difficile* toxins (Tcds) has been described by different authors [17–20, 24–26]. In the intrinsic pathway, TcdA or TcdB [17–19, 24, 25], increased levels of pro-apoptotic Bcl-2 members and/or decreased levels of anti-apoptotic Bcl-2 members, permeabilized mitochondria to release pro-apoptotic factors, such as cytochrome c, which triggers procaspase-9 activation, followed by downstream apoptotic effector activation [17–20, 24–26].

However, the mechanism by which apoptosis is activated by Tcds is more complex than that characterized by caspase activation alone, as highlighted by Nottrott et al., who showed that TcdA-induced apoptosis in HT-29 cells depended on the activation of both caspase-3 and non-caspase proteases, particularly cathepsins, since the cathepsin/calpain inhibitor N-acetyl-Leu-Leu-methioninal (ALLM) inhibited TcdA-induced apoptosis, but caspase-independent apoptosis was not observed, and because caspase-3 remains the central caspase responsible for apoptosis [22].

Until now, there have been no further studies on the role of non-caspase proteases in TcdA-induced apoptosis and no studies on the role of non-caspase proteases in apoptosis induced by TcdB.

The pre- and post-mitochondrial phases of caspase-dependent or caspase-independent apoptosis can be mediated by non-caspase proteases, and the best described non-caspase proteases involved in apoptosis are cathepsins and calpains [35–43]. Cathepsins are cysteine, aspartyl or serine proteases located in lysosomes. Cysteine protease cathepsins, the most abundant type, are involved in almost all lysosomal processes and several different cellular processes and can induce caspase-independent or partially caspase-dependent apoptosis [35, 42–44]. Calpains, non-lysosomal calcium (Ca^2+^)-dependent cysteine proteases, are frequently activated in caspase-independent or partially caspase-dependent apoptosis [36–41]. They are localized in the cytosol and possess specific tissue isoforms [39, 40]. In particular, the two ubiquitous isoforms are μ-calpain and m-calpain, which are activated by micromolar and millimolar levels of Ca^2+^, respectively [39, 40]. Both cathepsins and calpains share cell death substrates with caspases, including caspases themselves, members of the Bcl-2 family [Bcl-2–associated X protein (Bax), Bid], PARP, and cytoskeletal proteins, and thus provoke effects very similar to those induced by the activation of caspases only [35–43].

The importance of caspases in TcdB-induced apoptosis has been established in several cell types [4–8, 24–28], but the involvement of other types of proteases beyond the caspase family, such as cathepsins and calpains, which may also play a role in the execution of apoptosis in caspase-independent or partially caspase-dependent pathways [36–40, 42, 43], has not been investigated in apoptosis induced by TcdB, particularly in the apoptosis of EGCs induced by TcdB and TcdB + CKs.

TNF-α (and/or IFN-γ) can induce apoptosis not only by caspase activation mediated by the extrinsic pathway triggered by caspase-8 [30–33] but also by Ca^2+^ influx, which can activate calpains [36–40] and furthermore through cathepsin B activation after lysosomal destabilization [38, 42–46], which can alter the overall balance between the pro-apoptotic [(cysteine proteases, c-Jun N-terminal kinase (JNK) and reactive oxygen species (ROS)] and pro-survival [Nuclear factor-kappa B (NF-κB), or phosphatidylinositol 3-kinase (PI3K)/AKT] arms of the TNF-α signalling cascade, based on the above considerations [30–33, 45–52].

In this study, therefore, we wanted to investigate the possible apoptotic pathways induced by TcdB in EGCs in the absence and presence of CKs.

This study demonstrates for the first time that TcdB induces apoptosis in EGCs by activating three signalling pathways, the most relevant pathway being activated by calpains that occurs both in a caspase-dependent and independent manner, another being activated by caspases that occurs in a caspase-dependent manner and finally another being activated by cathepsin B that occurs in a caspase-3- and caspase-7-independent manner. These pathways are activated to different degrees in TcdB and TcdB + CKs especially as regards to signal transduction mediated by these families of proteases towards downstream effects (apoptosis). This study demonstrates that the apoptotic signalling effects of TcdB are enriched by synergism between TcdB + CKs and accompanied by the activities induced by TNF-α which helps to enhance the apoptotic effect of TcdB, which, because it blocks Rac1, prevents TNF-α from partially activating the anti-apoptotic signalling pathway.

The ability to activate three different families of proteases that activate three apoptotic signalling pathways enhanced by cytotoxic synergism with the pro-inflammatory cytokines TNF-α and IFN-γ could be a very important strategy adopted by *C. difficile* because a cell may display resistance to a specific apoptotic pathway but TcdB-mediated activation of different apoptotic pathways increases the likelihood of inducing apoptosis if the target cell possesses an intrinsic resistance to one or two of the three pathways.

## Materials and methods

### TcdB

TcdB, isolated from *C. difficile* strain VPI10463, was purchased from Enzo Life Sciences (BML-G150-0050; Farmingdale, NY), reconstituted to 200 µg/ml to prepare a stock solution, and stored as indicated in data sheet at − 80 °C before use in the experiments at a concentration of 0.1 ng/ml [9, 53].

### Cell culture and treatment with TcdB

Rat-transformed EGCs [EGC/PK060399egfr (ATCC CRL-2690)] [54], purchased from ATCC (Manassas, VA, USA), were cultured in Dulbecco’s modified Eagle’s medium (DMEM) with 10% FBS, 2 mM l-glutamine, 100 U/ml penicillin, and 100 μg/ml streptomycin (complete medium) at 37 °C with 5% CO_2_ for no more than 20 passages [9, 53].

For the inhibitor experiments, EGCs were or were not pre-treated for 1 h with 50 µM Boc-Asp(OMe)-fluoromethylketone (BAF, a broad-spectrum caspase inhibitor; ALX-260-071 Enzo Life Sciences) [9, 39], 0.02 µM, 0.2 µM, 0.5 µM, 1 µM, 2 µM, 10 µM, 25 µM and 50 µM Q-Val-Asp-Oph, (Q-VD-OPh; a broad-spectrum caspase inhibitor; 1170 BioVision) [55–58], 2 µM, 10 µM, and 20 µM Z-Asp-Glu-Val-Asp-fluoromethylketone (Z-DEVD-fmk; a caspase-3- and caspase-7-specific inhibitor, ALX-260–141 Enzo Life Sciences) [9, 17, 20, 21, 24, 28, 39, 59], 20 µM Z-Ile-Glu(OMe)-Thr-Asp(OMe)-fluoromethylketone (Z-IETD-fmk; a specific inhibitor of caspase-8, ALX-260–073 Enzo Life Sciences) [17, 20, 21, 24], 100 μM 3-(4-iodophenyl)-2-mercapto-2-propenoic acid (PD150606, a selective inhibitor of µ- and m-calpain directed to the Ca^2+^-binding site; 1906 BioVision) [39, 60–62], 10 µM, 5 µM, and 1 µM [L-3-*trans*-(propylcarbamoyl)oxirane-2-carbonyl]-l-isoleucyl-l-proline methyl ester (CA-074Me, a specific inhibitor of cathepsin B; 205531 Millipore Corp., USA) [39, 43], 40 µM perifosine [(an AKT inhibitor; #14240 Cell Signaling Technology (CST), Beverly, MA, USA)] [53, 63, 64] or 10 µM SP600125 (a JNK inhibitor, #8177 CST) [10, 53] and then exposed to TcdB at 0.1 ng/ml for 1.5 h at 37 °C and 5% CO_2_. After this time, the cells were or were not stimulated with 50 ng/ml TNF-α (#300-01A PeproTech, Rocky Hill, NJ, USA) and 50 ng/ml IFN-γ (#400-20 PeproTech) for 24 h at 37 °C in 5% CO_2_. At 24 h the control and treated EGCs were detached as described above and washed, and cell viability and total cell number were determined by a trypan blue (Sigma) dye-exclusion assay. After evaluation of the viability and total number of cells, the control and treated EGCs were (a) aliquoted at 0.5 × 10^6^ in 12 × 75-mm tubes, after which the percentage of apoptosis was evaluated by flow cytometry, and (b) used to prepare whole-cell lysates [9, 53].

For experiments with combinations of inhibitors, EGCs were or were not pre-treated for 1 h with Z-DEVD-fmk (2 µM), PD150606 (100 µM), CA-074Me (10 µM), or combination of Z-DEVD-fmk (2 µM) plus PD150606 (100 µM), or combination of Z-DEVD-fmk (2 µM) plus CA-074Me (10 µM), were or were not exposed to TcdB (0.1 ng/ml) for 1.5 h, and were or were not stimulated with TNF-α (50 ng/ml) plus IFN-γ (50 ng/ml) (CKs). Cells from all experimental conditions were recovered at 24 h as above described and used for evaluation of the percentage of apoptosis by flow cytometry.

For kinetics experiments with inhibitors, EGCs were or were not pre-treated for 1 h with BAF (50 µM), PD150606 (100 µM), Q-VD-OPh (2 µM) and CA-074Me (10 µM) were or were not exposed to TcdB (0.1 ng/ml) for 1.5 h, and were or were not stimulated with TNF-α (50 ng/ml) plus IFN-γ (50 ng/ml) (CKs). Cells from all experimental conditions were recovered at 24 h, 48 h, and 72 h as above described and used for evaluation of the percentage of apoptosis by flow cytometry.

The inhibitors were maintained during the course of all experiments.

Inhibitors were resuspended with DMSO and before the use diluted in complete medium to obtain a final concentration of DMSO of 0.1% [BAF (50 µM), PD150606 (100 µM), Z-IETDfmk (20 µM) and CA-074Me (10 µM)] and 0.02% [for all the other inhibitors and concentrations used]. We have conducted experiments to evaluate the effects of DMSO alone, both on EGCs non-treated and treated with TcdB or TcdB + CKs using the final concentrations of DMSO 0.1% and 0.02%. The results obtained with all the concentrations of DMSO used show that in control, TcdB and TcdB + CKs treated EGCs there were not significant variations in cell proliferation, total cell number, and cell viability determined by a trypan blue dye-exclusion assay (data not shown), and on the cell cycle evaluated by flow cytometry (data not shown). Further, in cells treated with TcdB and TcdB + CKs, there was no significant effect also on apoptosis (data not shown). Western blot analysis confirms that the activation pattern of caspase-3 induced by TcdB and TcdB + CKs does not undergo a significant variation (data not shown), as well as there are no variations in the non-treated cells (data not shown).

For experiments with Ca^2+^ channel inhibitors or Ca^2+^ chelators, EGCs were or were not pre-treated for 30 min with 1 µM nifepidine (a specific inhibitor of L-type voltage-dependent Ca^2+^ channels; Enzo Life Sciences) [65, 66], 20 µM nickel(II) chloride (NiCl_2_) (a specific inhibitor of low-voltage-activated T-type Ca^2+^ channels; Jena Bioscience) [65], 1 mM BAPTA-AM (Thermo Fisher Scientific), or 1 mM EGTA (Sigma). The EGCs then were or were not treated with TcdB and were or were not stimulated with TNF-α and IFN-γ as described above.

### Evaluation of cell cycle and apoptosis by flow cytometry

The control and treated EGCs were recovered at 24 h or in some experiments also at 48 h and 72 h and analysed by flow cytometry to evaluate the DNA content and detect apoptosis and cell cycle changes [9, 53].

To this, the cell pellets were resuspended in 1 ml of a hypotonic fluorochrome solution (50 μg/ml PI in 0.1% sodium citrate plus 0.1% Triton X-100) [9, 39, 53]. The samples were incubated at 4 °C in the dark for 2 h, and the PI fluorescence of each nucleus was evaluated with an EPICS XL-MCL flow cytometer (Beckman Coulter, FL, USA) [9, 39, 53, 59]. The data were processed with an Intercomp computer and analysed with EXPO32 software (Beckman Coulter) [9, 39, 53, 59]. The percentage of apoptotic cells (hypodiploid DNA content) was determined with EXPO32 software (Beckman Coulter) [9, 39, 53, 59]. Flow cytometry analyses were repeated three-six  times in independent experiments. DNA fluorescence flow cytometric profiles of one experiment representative of three-five, and graph showing the mean ± standard deviation of percentage hypodiploid nuclei obtained in three-six different experiments are shown. The data were analysed as described in Statistical analysis section. The cell cycle was analysed by measuring DNA-bound PI fluorescence in the orange-red fluorescence channel (FL2) with linear amplification. The percentage of cells in each cell cycle phase was analysed with ModFit software (Verity Software House, Topsham, ME, USA) [9, 53]. Flow cytometry analyses were repeated in six independent experiments. The data are the mean ± standard deviation of percentage of cells in each cell cycle phase obtained in six different experiments. The data were analysed as described in Statistical analysis.

### Apoptosis evaluation by Annexin V/PI assay and flow cytometry

To distinguish the early apoptosis from the late apoptosis, the apoptosis was assessed also by flow cytometric analysis of annexin V-fluorescein isothiocyanate (FITC)/PI-stained cells assay using a commercial kit (Beckman Coulter) according to the manufacturer's instructions and then the percentage of cells in the different phases was evaluated. To this, the control and treated EGCs recovered at 24 h were pelleted and resuspended in binding buffer and co-stained with FITC-conjugated annexin V plus PI. After incubation at room temperature for 15 min, the stained cells were analysed by flow cytometry (EPICS-XL-MCL) with an Intercomp computer and EXPO32 software. The percentage of cells Annexin V+, PI+, AnnexinV+/PI+ and AnnexinV−/PI− was determined with EXPO32 software (Beckman Coulter) [9, 39, 53, 59]. Flow cytometry analyses were repeated three times in independent experiments. Fluorescence flow cytometric profiles of one experiment representative of three, and graph showing the mean ± standard deviation of percentage of cells obtained in three different experiments are shown. The data were analysed as described in Statistical analysis.

### Protein extraction and Western blot analysis

At 24 h, control EGCs and treated EGCs were lysed with 100 μl of modified radioimmunoprecipitation assay lysis buffer containing protease and phosphatase inhibitors (Sigma-Aldrich) as previously described [9, 53].

The protein content was determined with a standard Bradford protein assay (Bio-Rad Laboratories, Milan, Italy). Proteins (20 µg) were separated by 7.5%, 10%, and 12% sodium dodecyl sulphate–polyacrylamide gel electrophoresis (SDS-PAGE) [9, 53] and transferred to nitrocellulose membranes using Trans-Blot Turbo transfer system (Bio-Rad). Then, the filters were blocked and incubated overnight at 4 °C with the following primary antibodies (Abs) [9, 53]: rabbit-specific polyclonal Abs specific for caspase-3 (1:1000; #9662 CST), caspase-7 (1:1000; #9492 CST), PARP (1:1000; #9542 CST), caspase-9 (1:1000; #9506 CST), phospho-JNK (Thr183/Tyr185; pJNK; 1:1000; #4668 CST), phospho-AKT (Ser473; pAKT; 1:1000; #4060 CST) and AKT (1:1000; #4691 CST), rabbit polyclonal Ab specific for nuclear factor of kappa light polypeptide gene enhancer in B-cells inhibitor alpha (IkBα 1:1000; E-AB-31839 Elabscience); rabbit polyclonal Ab specific for Bid (1:1000; 0.25 μg/ml final concentration; 10988-1-AP Proteintech); and mouse monoclonal Ab specific for α‐spectrin (nonerythroid; 1:2000, 0.05 μg/ml final concentration; MAB1622 Chemicon International, Temecula, CA). Signals were detected with horseradish peroxidase (HRP)-conjugated mouse anti-IgG (CST; 1:1000 for target proteins and 1:5000 for housekeeping proteins; #7076 CST) and rabbit anti-IgG secondary Abs (1:2000; #7074 CST) and an enhanced chemiluminescence system (GE Healthcare, Milan, Italy) [9, 53]. The membranes were stripped with Restore Plus solution (Thermo Scientific, USA) for 15 min at 37 °C, washed, and re-probed. A mouse monoclonal Ab specific for β-actin (clone AC-15; 1:20,000; 0.15 μg/ml final concentration; A5441, Sigma, St. Louis, MO) or for β-tubulin (clone TUB 2.1; 1:200; 15.5 μg/ml final concentration; T4026 Sigma) were used as loading control [9, 53]. The results of one experiment representative of three independent experiments are shown. Densitometric analysis was performed after scanning with Quantity One software (Bio-Rad, Milan, Italy). The results are expressed as arbitrary densitometric units (DU) relative to the density of β-actin, or β-tubulin or AKT from three independent experiments.

### Statistical analysis

All data were expressed as mean ± SD, GraphPad Prism 9.0.0 software was used to make statistical charts. Comparisons of multiple groups were analyzed using one-way ANOVA followed by Tukey’s post hoc test for evaluating the significance of the differences between the groups. *P* values = or less than 0.05 were defined as statistically significant.

## Results

Previously, we showed that TcdB induces apoptosis in EGCs and that this apoptosis significantly increased following stimulation with the pro-inflammatory cytokines TNF-α and IFN-γ (CKs) [9]. We also demonstrated that TcdB-induced EGC apoptosis is caspase-dependent and mitochondria-independent and that stimulation with CKs increased the activation of caspase-3, caspase-7, caspase-9 and PARP induced by TcdB without significant effects on the expression of Bax or B-cell lymphoma-x long isoform (Bcl-X_L_) [9], indicating that the CKs enhanced susceptibility to apoptosis induced by TcdB mainly by increasing the activation of these caspases [9].

However, in this study we analysed the roles of other key pathways other than mediated by caspases involved in transduction of pro-apoptotic signals, cathepsin and calpain pathways, and those of key anti-apoptotic signals involved in cell survival in EGCs treated with both TcdB and TcdB + CKs, in the light of the following: (a) TcdA can activate cell death pathways mediated by cathepsins/calpains beyond those regulated by caspases [22], but whether such apoptotic pathways are also activated in TcdB-induced EGC apoptosis and in CK-mediated enhancement of TcdB-induced EGC apoptosis is unknown. (b) It is unknown whether calpain and/or cathepsin activation would antagonize or synergize with the caspase pathway in TcdB apoptosis. (c) TcdB and TNF-α can induce Ca^2+^ influx [24, 65–67], which could activate calpains [36–40]. (d) TNF-α can activate apoptosis by receptor-mediated pathway by recruiting and activating procaspase-8, triggering the activation of downstream effectors [30–33]. (e) TNF-α can induce apoptosis by not only caspase-8 but also cathepsin B [38, 42–46]. (f) TNF-α-induced apoptosis can also be caused by a change in the balance between the pro-apoptotic (caspases, cathepsins, JNK, ROS) and cell survival arms (NF-κB, or PI3K/AKT) of the TNF-α signalling cascade [32, 33, 45–52]. (g) Finally, the inhibition of Rac1 could increase sensitivity to TNF-α-mediated apoptosis [68, 69].

## Role of pro-apoptotic signalling pathways in EGC apoptosis induced by TcdB and by TcdB plus TNF-α and IFN-γ (CKs)

### *TcdB and TcdB* + *CKs induce apoptosis, alterations in cell proliferation and cell-cycle arrest in EGCs*

To analyse the roles of key pathways involved in transduction of pro-apoptotic signals in EGC apoptosis induced by TcdB and by TcdB + CKs, we chose the lowest concentration of TcdB that induces apoptosis in EGCs (0.1 ng/ml) and synergizes with CKs, and 24 h of in vitro treatment, in which the maximum effects on both apoptosis and caspase activation were observed [9].

So, we preliminarily evaluated: (a) the percentage of apoptotic cells (hypodiploid DNA content) by flow cytometry, (b) cell viability and total cell number by trypan blue staining of cells, and (c) cell cycle distribution by flow cytometry in EGCs treated with TcdB alone or with TcdB + CKs as described in the Materials and methods section.

**Fig. 1:**
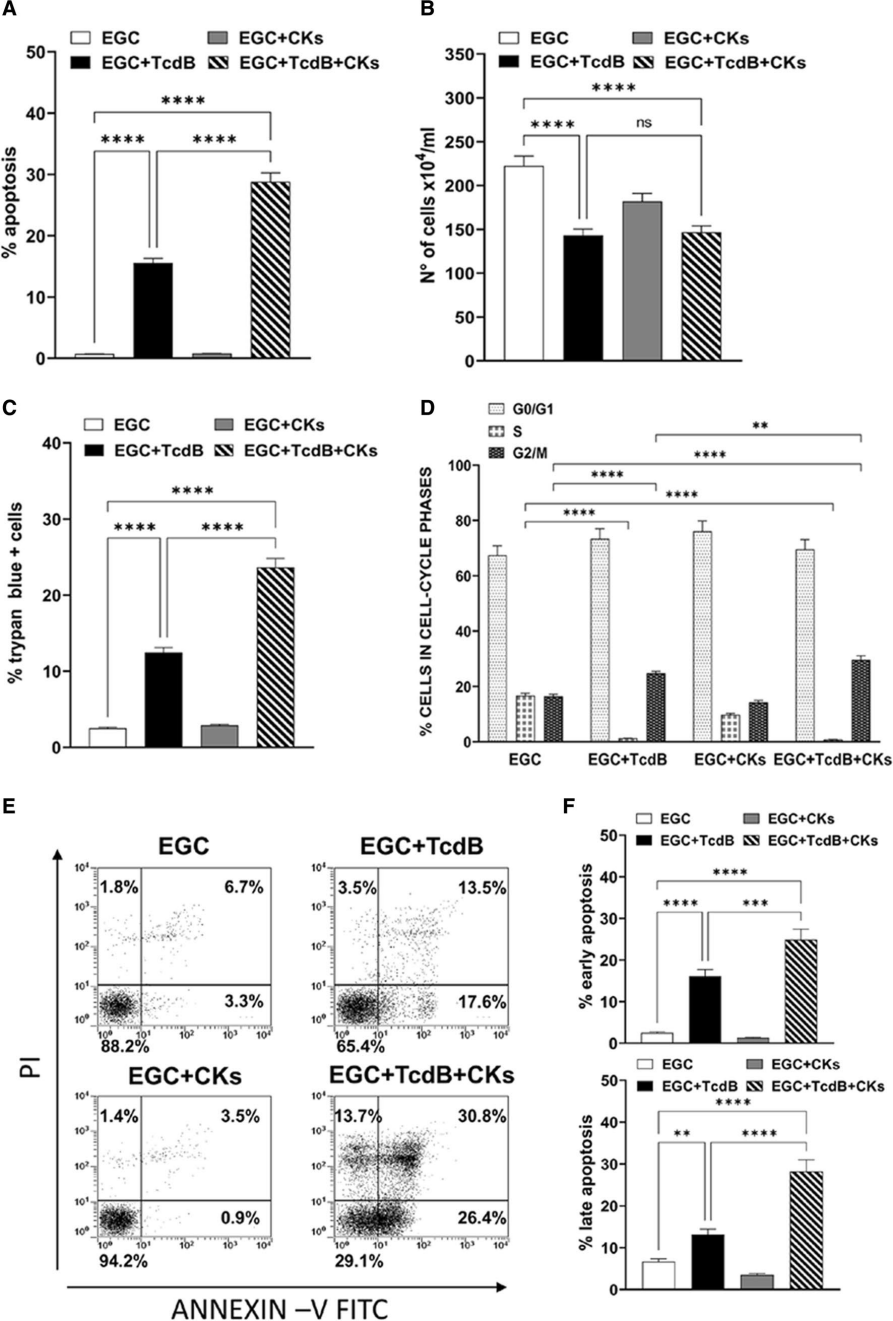
Effect of TcdB and TcdB +CKs on EGC apoptosis, cell viability, cell growth and cell cycle phases. EGCs were or were not exposed to TcdB (0.1 ng/ml) for 1.5 h and were or were not stimulated with TNF-α (50 ng/ml) plus IFN-γ (50 ng/ml) (CKs). Cells from all experimental conditions were recovered at 24 h and was determined: **A** apoptosis measuring the percentage of hypodiploid nuclei by flow cytometry; the total cell number (**B**) or the percentage of trypan blue + cells (**C**), by trypan blue dye-exclusion assay; **D** the percentages of cells in the cell cycle phases by flow cytometry with ModFit software; **E, F** apoptosis measuring the percentage of both Annexin V+ cells and Annexin V+/PI+ cells by flow cytometry. In **A–D** The data are the mean ± standard deviation of six experiments. In **E** are shown the flow cytometric profiles with percentages of cells + for each condition of one experiment representative of three, and in **F** are reported the graphs showing the mean ± standard deviation of percentage of Annexin V+ cells or Annexin V+/PI+ cells in three different experiments. **A–D**, **F** Statistical analysis was performed by one-way ANOVA and Tukey’s multiple comparisons test. *P < 0.05, **P < 0.01, ***P < 0.001, ****P < 0.0001, ns *P* > 0.05

The results obtained indicate that 0.1 ng/ml of TcdB alone induces about 15% apoptosis in EGCs (Fig. 1A) and TcdB + CKs doubled the percentage of apoptotic cells, indeed we found approximately 28% (Fig. 1A) while no apoptosis was detected in controls and CK-treated EGCs (Fig. 1A). The total number of EGCs treated with TcdB alone and TcdB + CKs was reduced by approximately 34% with respect to non-treated EGCs (Fig. 1B). Regarding the percentage of trypan blue + cells, we found a 12.5% of trypan blue + cells after administration of TcdB alone and 23.6% after TcdB + CKs (Fig. 1C). Moreover, we found an accumulation of cells in the 4 N peak of flow cytometry (cells in the G2/M phase of the cell cycle and bi-nucleated cells in G1 phase) accompanied by a decrease in the number of cells in the S phase both after TcdB alone and TcdB + CKs (Fig. 1D). At 24 h, the percentage of control EGCs non stimulated and stimulated with CKs in the 4 N peak of flow cytometry (cells in the G2/M phase of the cell cycle and bi-nucleated cells in G1 phase) was approximately 16.4% and 14.3%, respectively, and the percentage increased to 25.2% and 29.6%, respectively after administration of TcdB alone and TcdB + CKs (Fig. 1D). Flow cytometry showed that there are TcdB-treated cells with not only 2 N and 4 N, but also with higher numbers of chromosome content. This is due to Rho inhibition which prevents cytokinesis but not cariogenesis, leading to bi- and tetra-nucleated cells. Therefore, this means that 4 N peak in flow cytometry does not necessarily means G2/M block but instead bi-nucleated cells in G1 phase then TcdB alone and TcdB + CKs induced a cell cycle arrest in G1 and G2/M phases.

Altogether these results demonstrate that TcdB alone and Tcd + CKs induce apoptosis with a reduction of both cell viability and total cell number. Further, the reduction of cell number observed between control and TcdB and control and TcdB + CKs treated EGCs is due to the growth inhibition of EGCs induced by TcdB alone or TcdB + CKs that was associated with the induction of cell cycle arrest in G1 and G2/M phases.

To distinguish the early apoptosis from the late apoptosis we assessed apoptosis also by Annexin V-FITC/PI assay and flow cytometric analysis. Flow cytometry of Annexin V/PI-stained cells shows the induction of early and late apoptosis upon TcdB and TcdB + CKs treatment (Fig. 1E, F). Upon treatment with TcdB alone, there was approximatively 17.6% of Annexin V+ cells, 13.5% of Annexin V+/PI+ cells and only a 3.5% of PI+ cells (Fig. 1E, F) while upon treatment with TcdB + CKs these values were strong increased with approximatively 26.4% of Annexin V + cells, 30.8% of Annexin V+/PI+ cells and 13.7% of PI+ cells (Fig. 1E, F). Therefore, these results, confirm that CKs enhanced susceptibility to EGC apoptosis induced by TcdB, as demonstrated by analysis of apoptosis measuring the percentage of hypodiploid DNA content by flow cytometry.

### Role of overall contribution of caspases

To investigate the role of caspases in TcdB- and TcdB + CK-induced EGC apoptosis in greater depth we first examine the role of initiator and effector caspases namely the overall contribution of caspases, analysing the effects of BAF (a pan-caspase inhibitor) [9, 39] on the percentage of apoptotic cells (hypodiploid DNA content) by flow cytometry, the activation of caspase-3 and caspase-7 (as measurement of upstream caspase activation) and PARP (as measurement of executioner caspase activation) by Western blot analysis in EGCs treated with TcdB and TcdB + CKs.

**Fig. 2:**
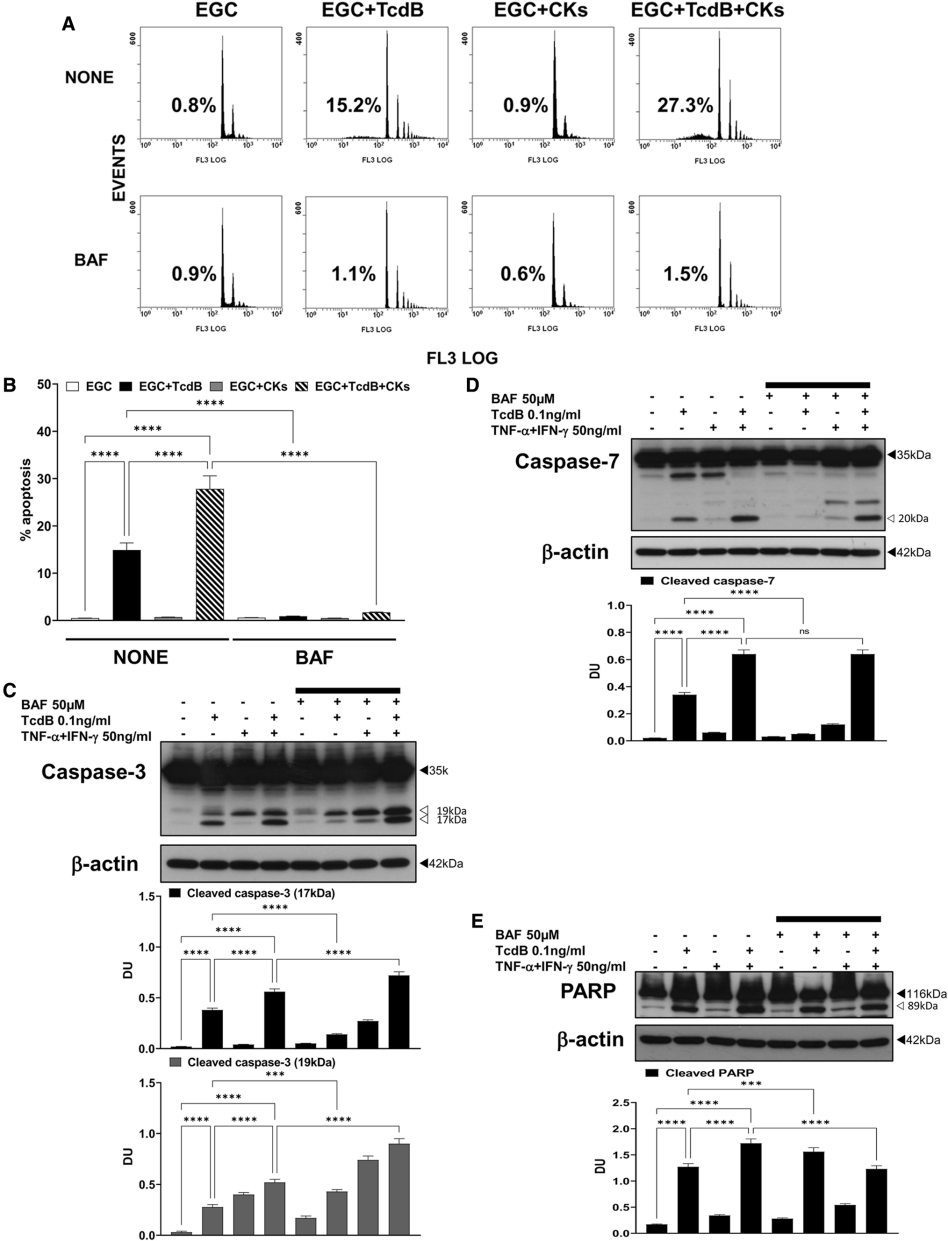
Pan-caspase inhibition with BAF prevented apoptosis induced by TcdB and TcdB + CKs but had different effects on caspase-3, caspase-7 and PARP activation after TcdB and TcdB + CK treatment. EGCs were or were not pre-treated for 1 h with BAF (50 µM), were or were not exposed to TcdB (0.1 ng/ml) for 1.5 h and were or were not stimulated with TNF-α (50 ng/ml) plus IFN-γ (50 ng/ml) (CKs). Cells from all experimental conditions were recovered at 24 h to evaluate apoptosis (**A**, **B**) and prepare whole-cell lysates for SDS-PAGE and Western blot analysis (**C–E**). **A**, **B** Apoptosis was evaluated by measuring the percentage of hypodiploid nuclei by flow cytometry. DNA fluorescence flow cytometric profiles with percentages of hypodiploid nuclei of one experiment representative of five (**A**), and graph showing the mean ± standard deviation of percentage of hypodiploid nuclei obtained in five different experiments (**B**) are shown. **C–E** Filters were probed with **C** anti-caspase-3, **D** anti-caspase-7 or **E** anti-PARP Abs. All filters were stripped and re-probed with anti-β-actin Ab. Blots are representative of three independent experiments. Intact protein (solid arrow) and active fragment (open arrow) are indicated. The graphs represent the mean ± standard deviation of densitometric analysis of cleaved caspase-3 (17 kDa), or cleaved caspase-3 (19 kDa), or cleaved caspase-7 (20 kDa), or cleaved PARP (89 kDa), relative to β-actin in three different experiments. **B–E** Statistical analysis was performed by one-way ANOVA and Tukey’s multiple comparisons test. *P < 0.05, **P < 0.01, ***P < 0.001, ****P < 0.0001, ns *P* > 0.05

**Fig. 3:**
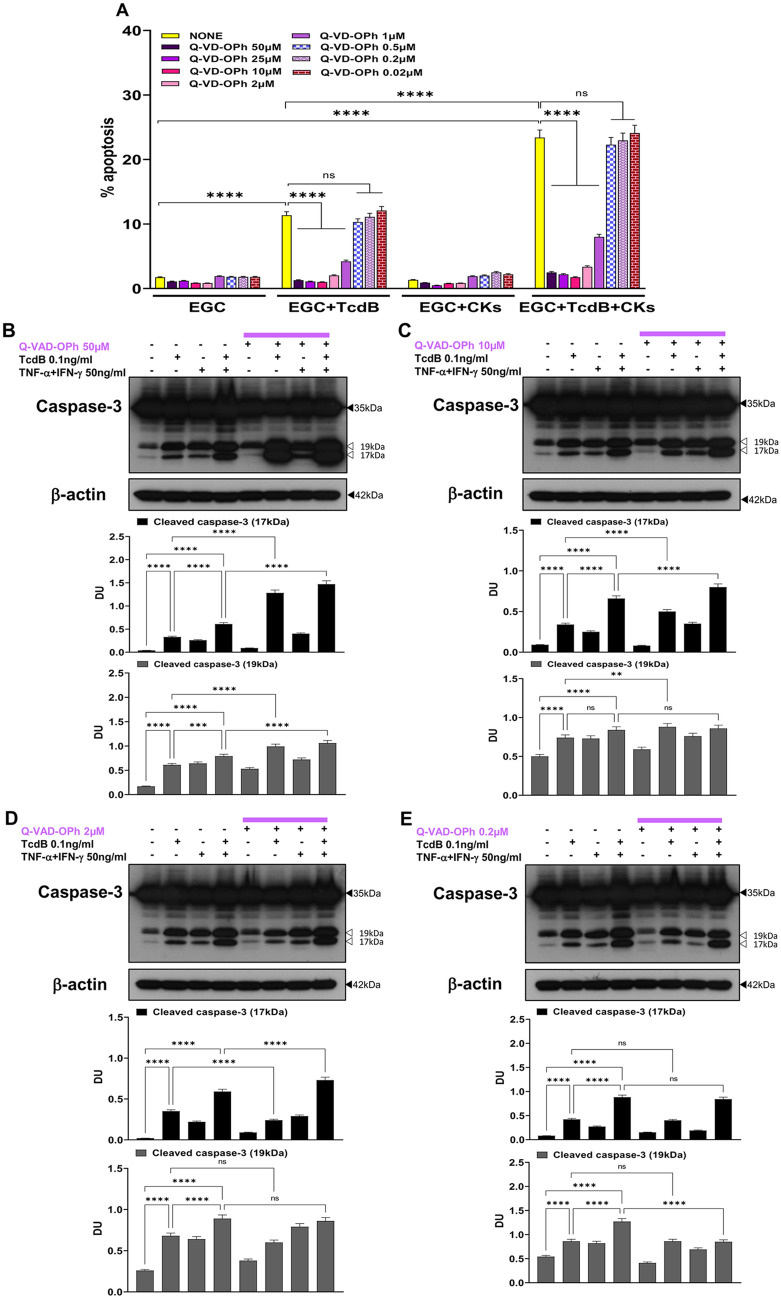
Effect of pan-caspase inhibition with Q-VD-OPh on apoptosis induced by TcdB and TcdB + CKs and on caspase-3 activation after TcdB and TcdB + CK treatment. EGCs were or were not pre-treated for 1 h with Q-VD-OPh at the doses indicated, were or were not exposed to TcdB (0.1 ng/ml) for 1.5 h and were or were not stimulated with TNF-α (50 ng/ml) plus IFN-γ (50 ng/ml) (CKs). Cells from all experimental conditions were recovered at 24 h to evaluate apoptosis (**A**) and prepare whole-cell lysates for SDS-PAGE and Western blot analysis (**B–E**). **A** Apoptosis was evaluated by measuring the percentage of hypodiploid nuclei by flow cytometry. Data are the mean ± standard deviation of percentage of hypodiploid nuclei obtained in three different experiments. **B–E** Filters were probed with anti-caspase-3 then were stripped and re-probed with anti-β-actin Ab. Blots are representative of three independent experiments. Intact protein (solid arrow) and active fragment (open arrow) are indicated. The graphs represent the mean ± standard deviation of densitometric analysis of cleaved caspase-3 (17 kDa), or cleaved caspase-3 (19 kDa), relative to β-actin in three different experiments. **A–E** Statistical analysis was performed by one-way ANOVA and Tukey’s multiple comparisons test. *P < 0.05, **P < 0.01, ***P < 0.001, ****P < 0.0001, ns *P* > 0.05

**Fig. 4:**
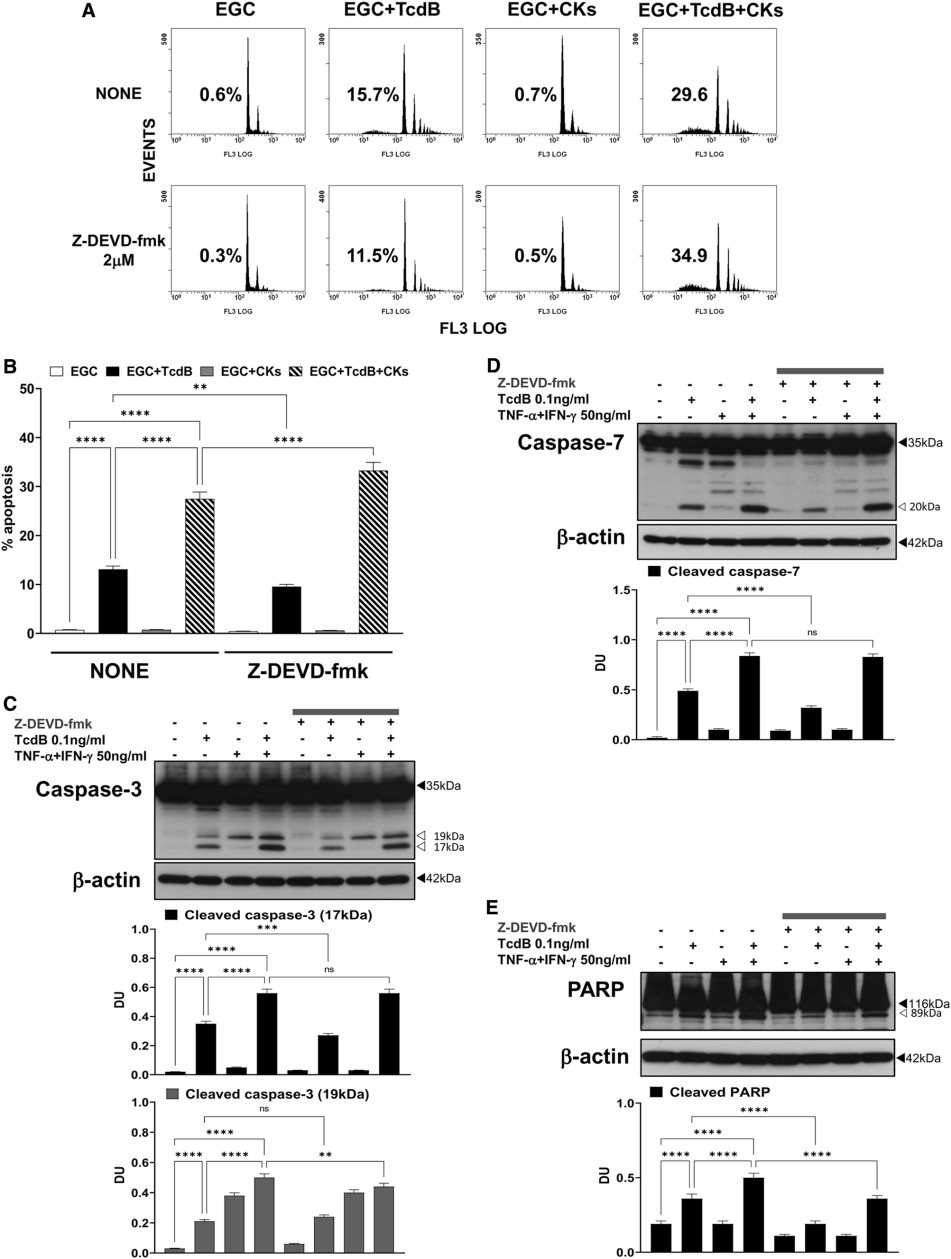
Effector caspase inhibition with Z-DEVD-fmk inhibited apoptosis induced by TcdB but not that induced by TcdB + CKs and had different effects on caspase-3, caspase-7 and PARP activation after TcdB and TcdB + CK treatment. EGCs were or were not pre-treated for 1 h with Z-DEVD-fmk (2 µM), were or were not exposed to TcdB (0.1 ng/ml) for 1.5 h and were or were not stimulated with TNF-α (50 ng/ml) plus IFN-γ (50 ng/ml) (CKs). Cells from all experimental conditions were recovered at 24 h to evaluate apoptosis (**A**, **B**) and prepare whole-cell lysates for SDS-PAGE and Western blot analysis (**C–E**). **A**, **B** Apoptosis was evaluated by measuring the percentage of hypodiploid nuclei by flow cytometry. DNA fluorescence flow cytometric profiles with percentages of hypodiploid nuclei of one experiment representative of five (**A**) and graph showing the mean ± standard deviation of percentage of hypodiploid nuclei obtained in five different experiments (**B**) are shown. Filters were probed with **C** anti-caspase-3, **D** anti-caspase-7 or **E** anti-PARP Abs. All filters were stripped and re-probed with anti-β-actin Ab. Blots are representative of three independent experiments. Intact protein (solid arrow) and active fragment (open arrow) are indicated. The graphs represent the mean ± standard deviation of densitometric analysis of cleaved caspase-3 (17 kDa), or cleaved caspase-3 (19 kDa), or cleaved caspase-7 (20 kDa), or cleaved PARP (89 kDa), relative to β-actin in three different experiments. **B–E** Statistical analysis was performed by one-way ANOVA and Tukey’s multiple comparisons test. *P < 0.05, **P < 0.01, ***P < 0.001, ****P < 0.0001, ns *P* > 0.05

**Fig. 5:**
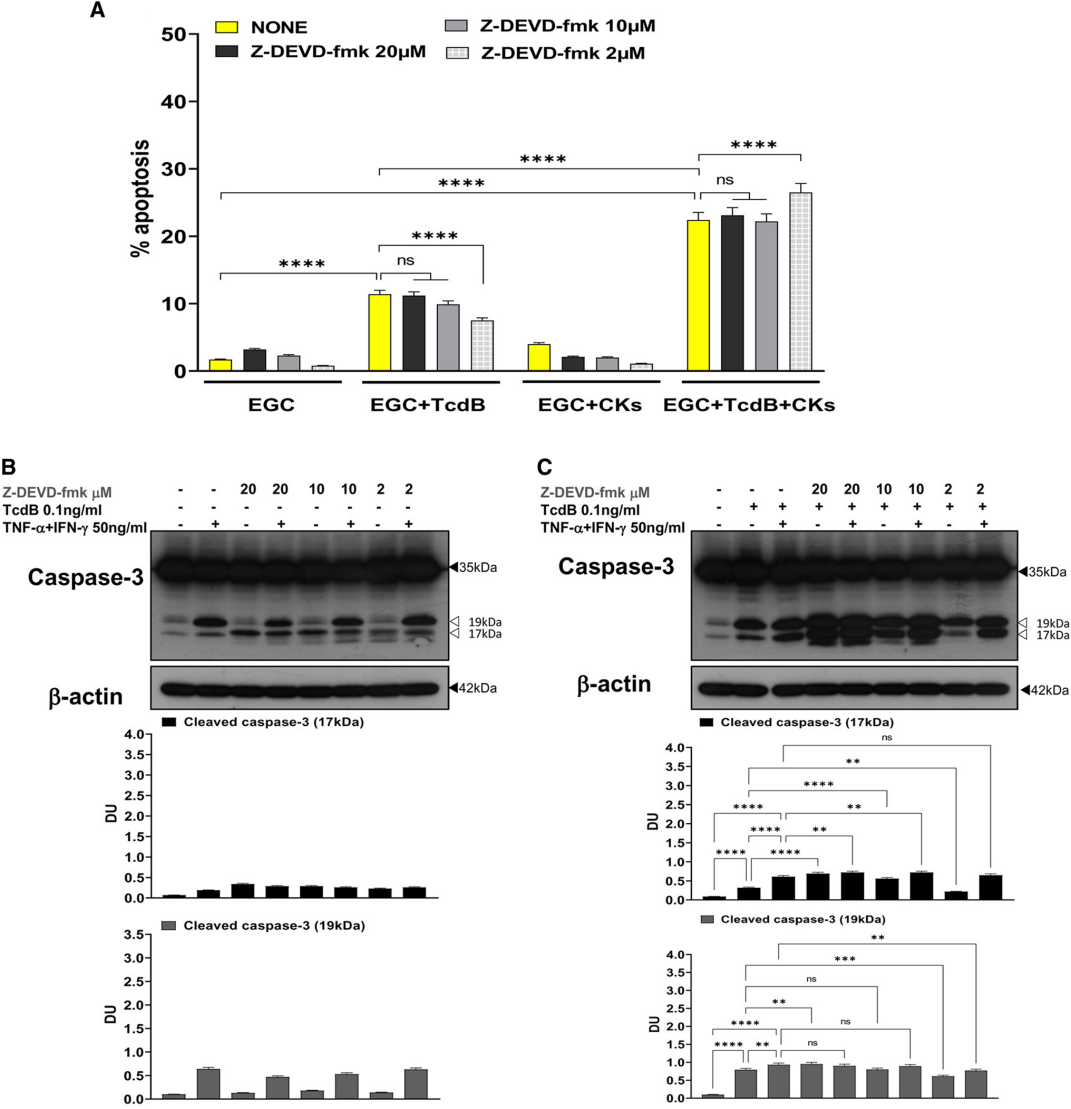
Effect of different doses of effector caspase inhibitor Z-DEVD-fmk in apoptosis induced by TcdB and TcdB + CKs and in caspase-3 activation. EGCs were or were not pre-treated for 1 h with Z-DEVD-fmk (20, 10 and 2 µM), were or were not exposed to TcdB (0.1 ng/ml) for 1.5 h and were or were not stimulated with TNF-α (50 ng/ml) plus IFN-γ (50 ng/ml) (CKs). Cells from all experimental conditions were recovered at 24 h to evaluate apoptosis (**A**) and prepare whole-cell lysates for SDS-PAGE and Western blot analysis (**B, C**). **A** Apoptosis was evaluated by measuring the percentage of hypodiploid nuclei by flow cytometry. Data are the mean ± standard deviation of percentage of hypodiploid nuclei obtained in three different experiments. **B**, **C** Filters were probed with anti-caspase-3 then were stripped and re-probed with anti-β-actin Ab. Blots are representative of three independent experiments. Intact protein (solid arrow) and active fragment (open arrow) are indicated. The graphs represent the mean ± standard deviation of densitometric analysis of cleaved caspase-3 (17 kDa) or cleaved caspase-3 (19 kDa) relative to β-actin in three different experiments. **A–C** Statistical analysis was performed by one-way ANOVA and Tukey’s multiple comparisons test. *P < 0.05, **P < 0.01, ***P < 0.001, ****P < 0.0001, ns *P* > 0.05

**Fig. 6:**
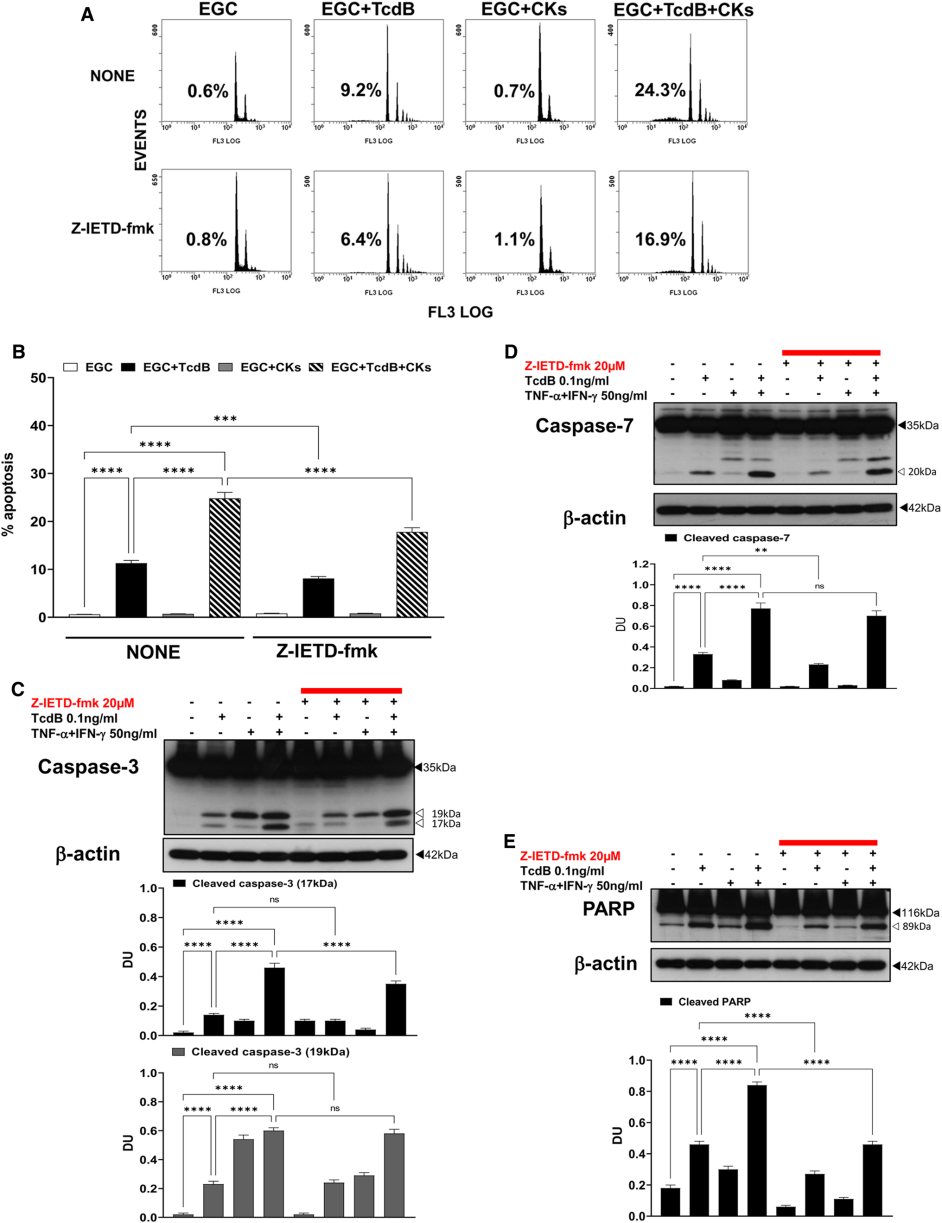
Caspase-8 inhibition with Z-IETD-fmk inhibited apoptosis and caspase-3, caspase-7 and PARP activation after TcdB and TcdB + CK treatment. EGCs were or were not pre-treated for 1 h with Z-IETD-fmk (20 µM), were or were not exposed to TcdB (0.1 ng/ml) for 1.5 h and were or were not stimulated with TNF-α (50 ng/ml) plus IFN-γ (50 ng/ml) (CKs). Cells from all experimental conditions were recovered at 24 h to evaluate apoptosis (**A**, **B)** and prepare whole-cell lysates for SDS-PAGE and Western blot analysis (**C–E**). **A**, **B** Apoptosis was evaluated by measuring the percentage of hypodiploid nuclei by flow cytometry. DNA fluorescence flow cytometric profiles with percentages of hypodiploid nuclei of one experiment representative of three (**A**) and graph showing the mean ± standard deviation of percentage of hypodiploid nuclei obtained in three different experiments (**B**) are shown. Filters were probed with **C** anti-caspase-3, **D** anti-caspase-7 or **E** anti-PARP Abs. All filters were stripped and re-probed with the anti-β-actin Ab. Blots are representative of three independent experiments. Intact protein (solid arrow) and active fragment (open arrow) are indicated. The graphs represent the mean ± standard deviation of densitometric analysis of cleaved caspase-3 (17 kDa), or cleaved caspase-3 (19 kDa), or cleaved caspase-7 (20 kDa), or cleaved PARP (89 kDa), relative to β-actin in three different experiments. **B–E** Statistical analysis was performed by one-way ANOVA and Tukey’s multiple comparisons test. *P < 0.05, **P < 0.01, ***P < 0.001, ****P < 0.0001, ns *P* > 0.05

**Fig. 7:**
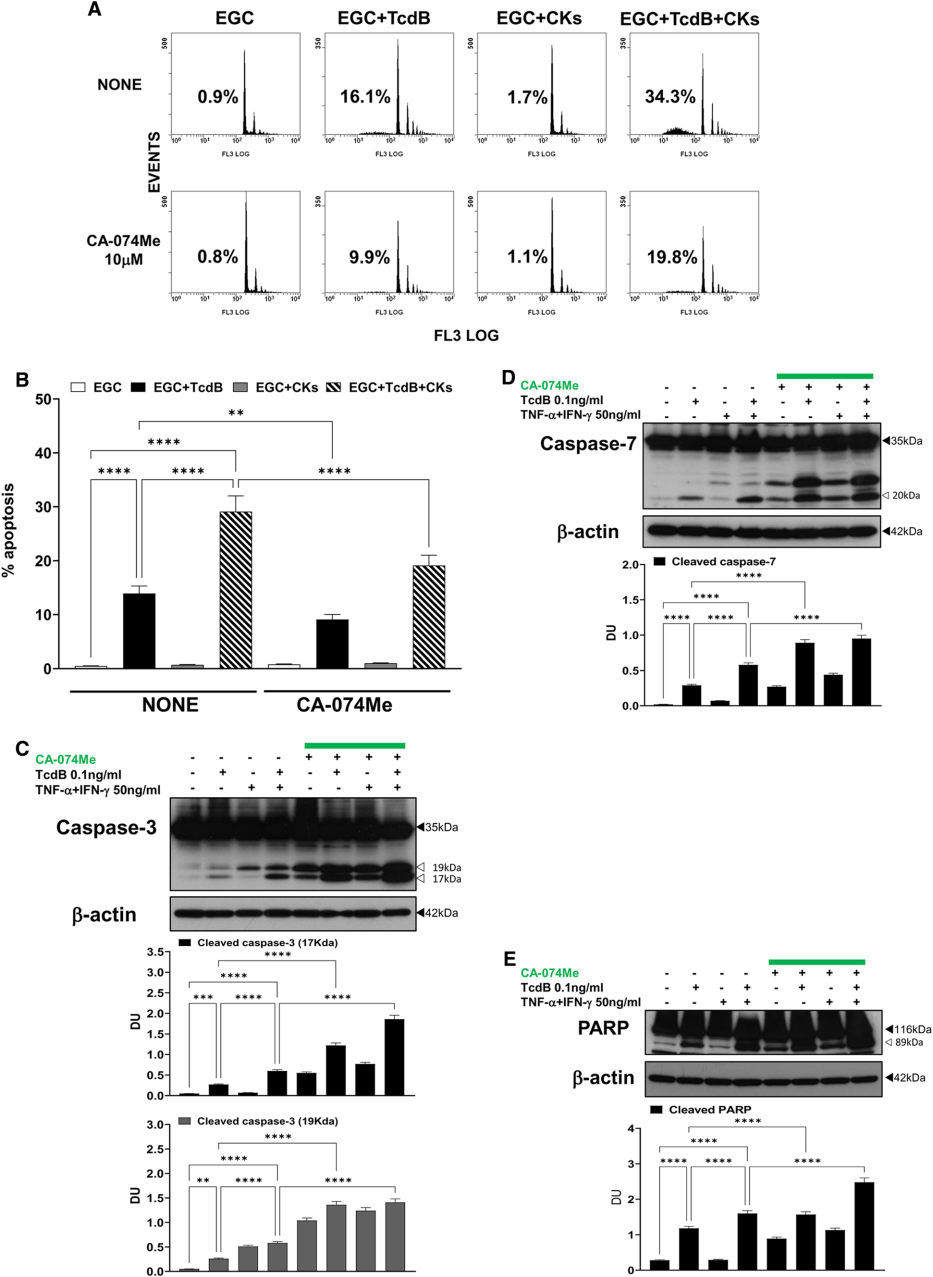
Cathepsin B inhibition with CA-074Me inhibited apoptosis induced by TcdB and TcdB + CKs but increased caspase-3, caspase-7 and PARP activation after TcdB and TcdB + CK treatment. EGCs were or were not pre-treated for 1 h with CA-074Me (10 µM), were or were not exposed to TcdB (0.1 ng/ml) for 1.5 h and were or were not stimulated with TNF-α (50 ng/ml) plus IFN-γ (50 ng/ml) (CKs). Cells from all experimental conditions were recovered at 24 h to evaluate apoptosis (**A**, **B**) and prepare whole-cell lysates for SDS-PAGE and Western blot analysis (**C–E**). **A**,** B** Apoptosis was evaluated by measuring the percentage of hypodiploid nuclei by flow cytometry. DNA fluorescence flow cytometric profiles with percentages of hypodiploid nuclei of one experiment representative of three (**A**) and graph showing the mean ± standard deviation of percentage of hypodiploid nuclei obtained in three different experiments performed in triplicate (**B**) are shown. Filters were probed with **C** anti-caspase-3, **D** anti-caspase-7 or **E** anti-PARP Abs. All filters were stripped and re-probed with the anti-β-actin Ab. Blots are representative of three independent experiments. Intact protein (solid arrow) and active fragment (open arrow) are indicated. The graphs represent the mean ± standard deviation of densitometric analysis of cleaved caspase-3 (17 kDa), or cleaved caspase-3 (19 kDa), or cleaved caspase-7 (20 kDa), or cleaved PARP (89 kDa), relative to β-actin in three different experiments. **B–E** Statistical analysis was performed by one-way ANOVA and Tukey’s multiple comparisons test. *P < 0.05, **P < 0.01, ***P < 0.001, ****P < 0.0001, ns *P* > 0.05

**Fig. 8:**
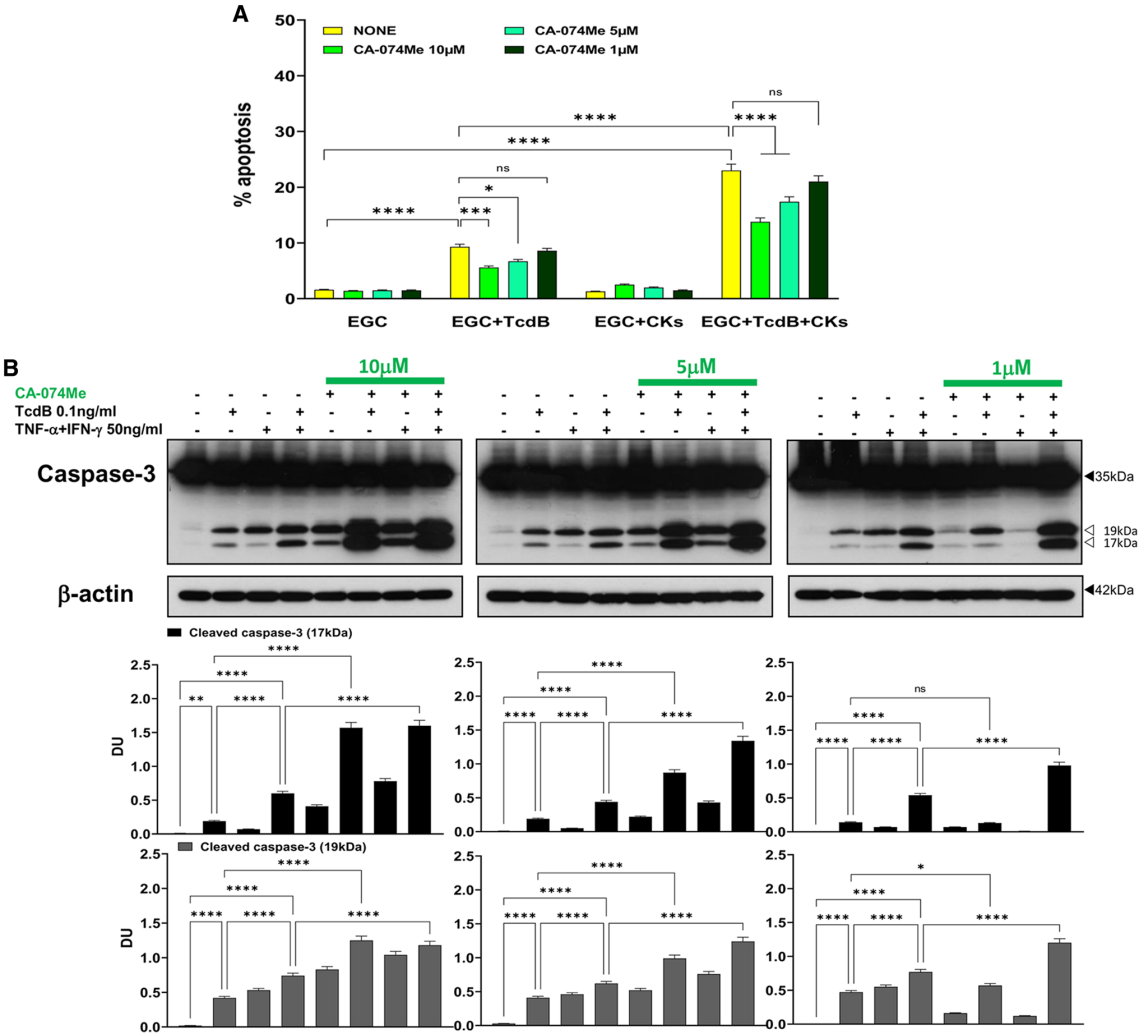
Effect of different doses of cathepsin B inhibitor CA-074Me in apoptosis induced by TcdB and TcdB + CKs and on caspase-3 activation. EGCs were or were not pre-treated for 1 h with CA-074Me (10, 5 and 1 µM), were or were not exposed to TcdB (0.1 ng/ml) for 1.5 h and were or were not stimulated with TNF-α (50 ng/ml) plus IFN-γ (50 ng/ml) (CKs). Cells from all experimental conditions were recovered at 24 h to evaluate apoptosis (**A**) and prepare whole-cell lysates for SDS-PAGE and Western blot analysis (**B**). **A** Apoptosis was evaluated by measuring the percentage of hypodiploid nuclei by flow cytometry. Data are the mean ± standard deviation of percentage of hypodiploid nuclei obtained in three different experiments. **B** Filters were probed with anti-caspase-3 then were stripped and re-probed with anti-β-actin Ab. Blots are representative of three independent experiments. Intact protein (solid arrow) and active fragment (open arrow) are indicated. The graphs represent the mean ± standard deviation of densitometric analysis of cleaved caspase-3 (17 kDa), or cleaved caspase-3 (19 kDa), relative to β-actin in three different experiments. **A–B** Statistical analysis was performed by one-way ANOVA and Tukey’s multiple comparisons test. *P < 0.05, **P < 0.01, ***P < 0.001, ****P < 0.0001, ns *P* > 0.05

**Fig. 9:**
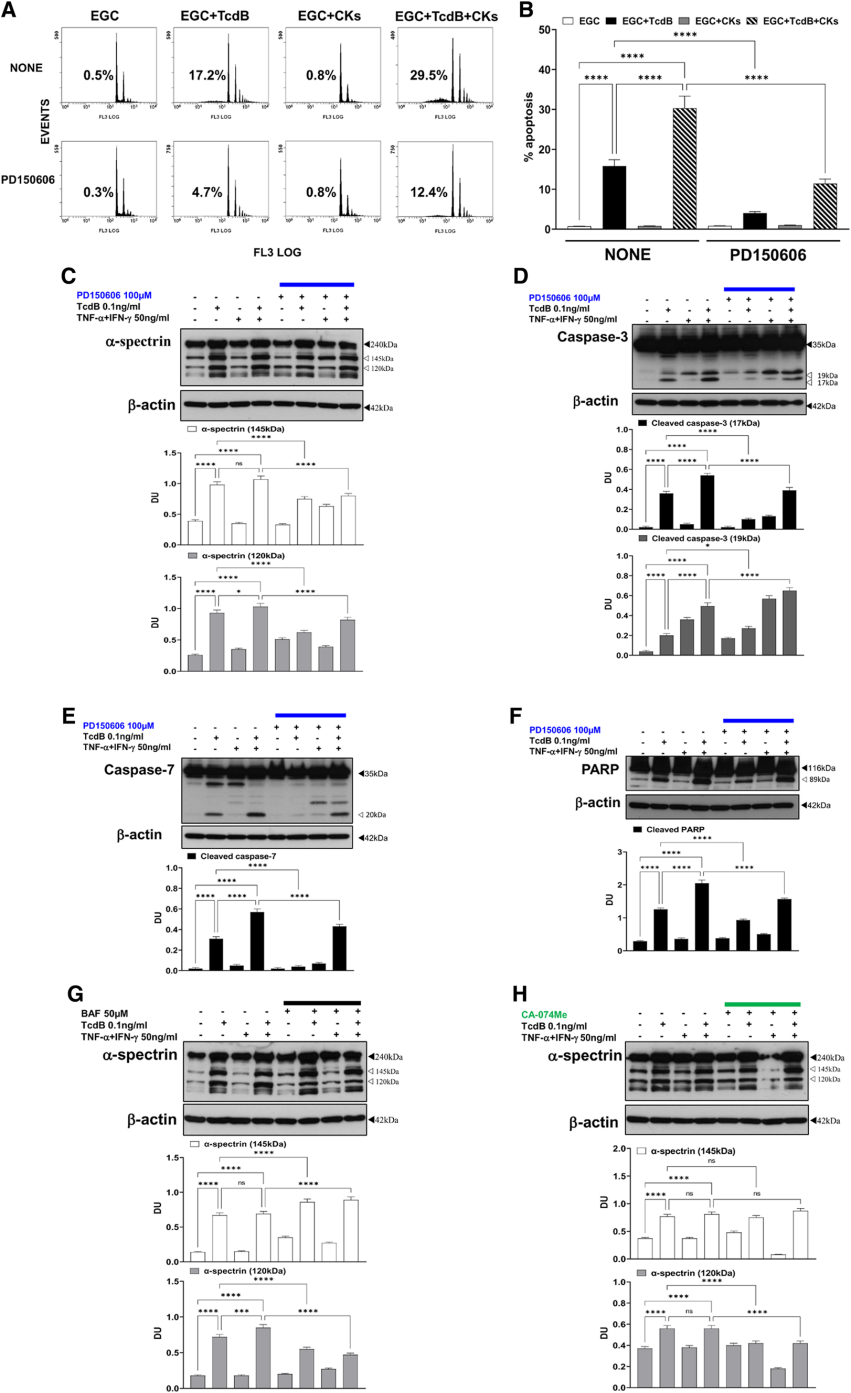
Calpain inhibition with PD150606 strongly inhibited apoptosis and α-spectrin cleavage*,* caspase-3, caspase-7 and PARP after TcdB and TcdB + CK treatment, while BAF and CA-074Me had different effects on α-spectrin cleavage. **A**, **B** EGCs were or were not pre-treated for 1 h with PD150606 (100 µM), were or were not exposed to TcdB (0.1 ng/ml) for 1.5 h and were or were not stimulated with TNF-α (50 ng/ml) plus IFN-γ (50 ng/ml) (CKs). Cells from all experimental conditions were recovered at 24 h to evaluate apoptosis. Apoptosis was evaluated by measuring the percentage of hypodiploid nuclei by flow cytometry. DNA fluorescence flow cytometric profiles with percentages of hypodiploid nuclei of one experiment representative of five (**A**) and graph showing the mean ± standard deviation of percentage of hypodiploid nuclei obtained in five different experiments performed in triplicate (**B**) are shown. **C–F** EGCs were or were not pre-treated for 1 h with PD150606 (100 µM), were or were not exposed to TcdB (0.1 ng/ml) for 1.5 h and were or were not stimulated with TNF-α (50 ng/ml) plus IFN-γ (50 ng/ml) (CKs). Cells from all experimental conditions were recovered at 24 h to prepare whole-cell lysates for SDS-PAGE and Western blot analysis. The filters were probed with **C** anti-α-spectrin, **D** anti-caspase-3, **E** anti-caspase-7 or **F** anti-PARP Abs. All filters were stripped and re-probed with the anti-β-actin Ab. Blots are representative of three independent experiments. Intact protein (solid arrow) and active fragment (open arrow) are indicated. The graphs represent the mean ± standard deviation of densitometric analysis of cleaved α-spectrin (145 kDa), of cleaved α-spectrin (120 kDa), cleaved caspase-3 (17 kDa), or cleaved caspase-3 (19 kDa), or cleaved caspase-7 (20 kDa), or cleaved PARP (89 kDa), relative to β-actin in three different experiments. EGCs were or were not pre-treated for 1 h with BAF (50 µM) (**G**) or with CA-074Me (10 µM) (**H**), were or were not exposed to TcdB (0.1 ng/ml) for 1.5 h and were or were not stimulated with TNF-α (50 ng/ml) plus IFN-γ (50 ng/ml). Cells from all experimental conditions were recovered at 24 h to prepare whole-cell lysates for SDS-PAGE and Western blot analysis. The filters were probed with anti-α-spectrin and then stripped and re-probed with anti-β-actin Ab (**G,** **H**). Blots are representative of three independent experiments. Intact protein (solid arrow) and active fragment (open arrow) are indicated. The graphs represent the mean ± standard deviation of densitometric analysis of cleaved α-spectrin (145 kDa), or cleaved α-spectrin (120 kDa) relative to β-actin in three different experiments. **B–H** Statistical analysis was performed by one-way ANOVA and Tukey’s multiple comparisons test. *P < 0.05, **P < 0.01, ***P < 0.001, ****P < 0.0001, ns *P* > 0.05

**Fig. 10:**
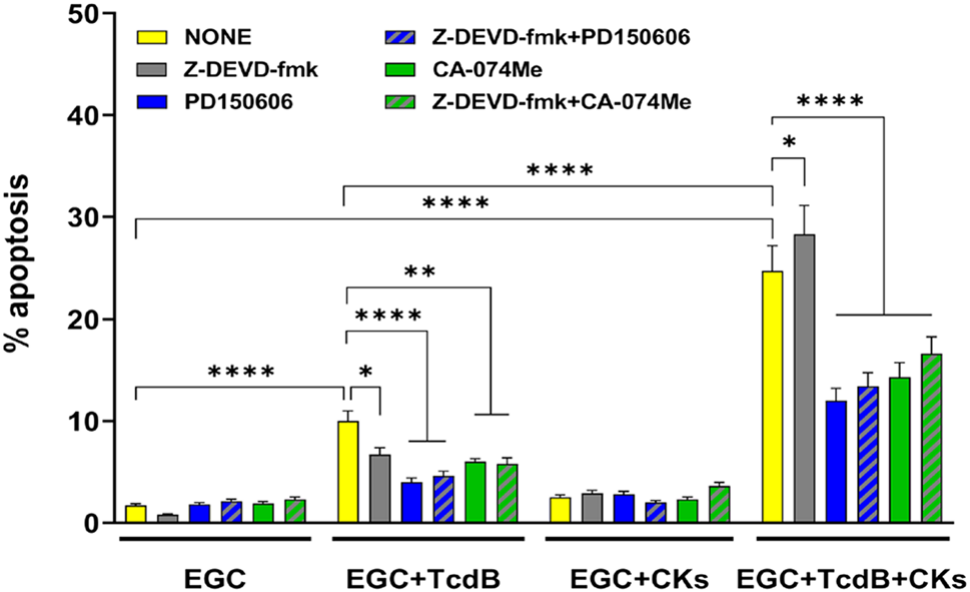
Combination of Z-DEVD-fmk with PD150606 or CA-074Me does not induce a synergistic effect on apoptosis inhibition after TcdB and TcdB + CK treatment. EGCs were or were not pre-treated for 1 h with Z-DEVD-fmk (2 µM), PD150606 (100 µM), CA-074Me (10 µM), Z-DEVD-fmk (2 µM) plus PD150606 (100 µM), Z-DEVD-fmk (2 µM) plus CA-074Me (10 µM), were or were not exposed to TcdB (0.1 ng/ml) for 1.5 h, and were or were not stimulated with TNF-α (50 ng/ml) plus IFN-γ (50 ng/ml) (CKs). Cells from all experimental conditions were recovered at 24 h to evaluate apoptosis. Apoptosis was evaluated by measuring the percentage of hypodiploid nuclei by flow cytometry. Data are the mean ± standard deviation of percentage of hypodiploid nuclei obtained in three different experiments. Statistical analysis was performed by one-way ANOVA and Tukey’s multiple comparisons test. *P < 0.05, **P < 0.01, ***P < 0.001, ****P < 0.0001, ns *P* > 0.05

**Fig. 11:**
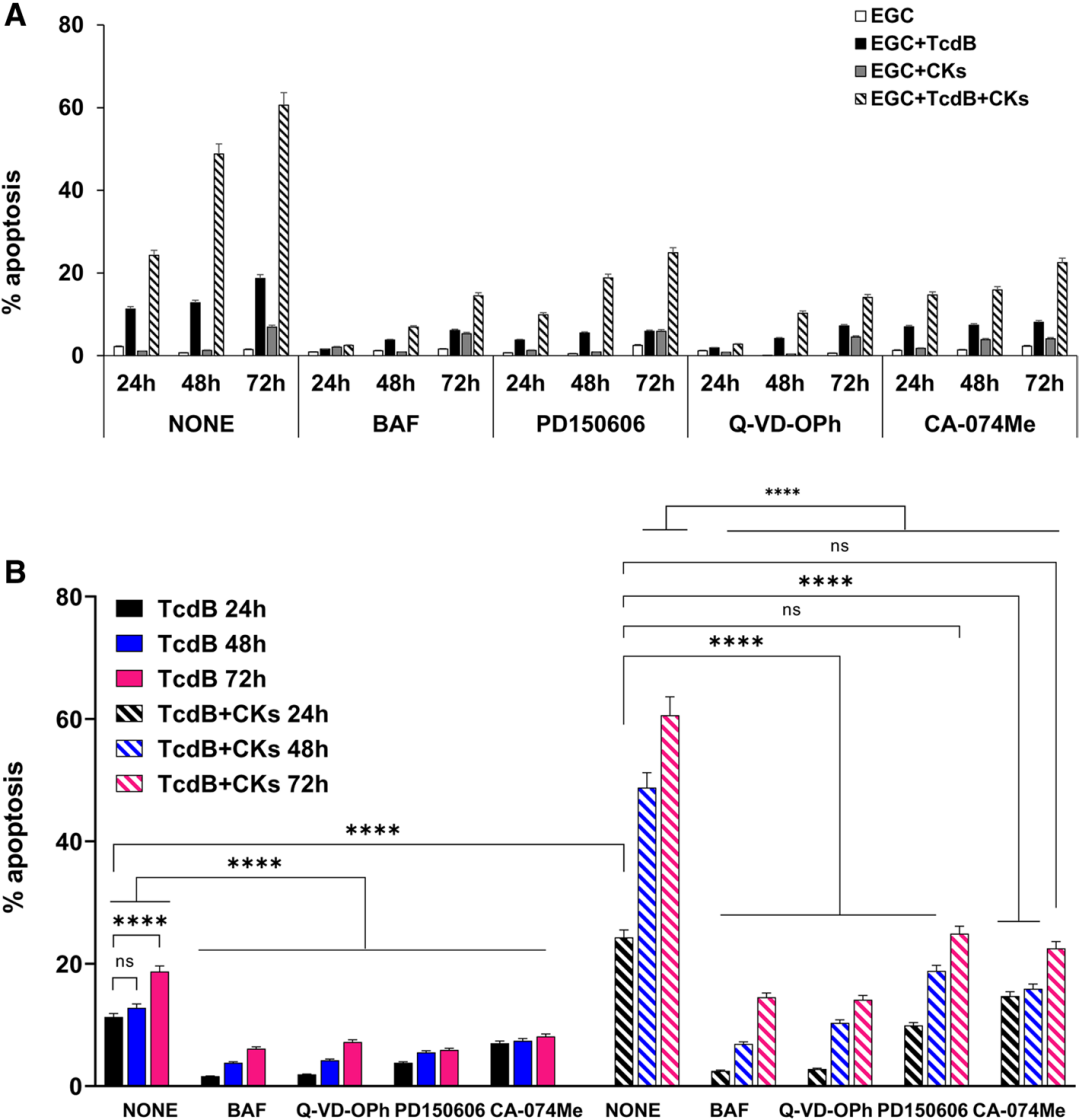
Effect of BAF, Q-VD-OPh, PD150606 and CA-074Me on EGC apoptosis induced by TcdB and TcdB + CKs at longer timepoints. EGCs were or were not pre-treated for 1 h with BAF (50 µM), PD150606 (100 µM), Q-VD-OPh (2 µM) and CA-074Me (10 µM) were or were not exposed to TcdB (0.1 ng/ml) for 1.5 h, and were or were not stimulated with TNF-α (50 ng/ml) plus IFN-γ (50 ng/ml) (CKs). Cells from all experimental conditions were recovered at 24 h, 48 h, and 72 h to evaluate apoptosis. Apoptosis was evaluated by measuring the percentage of hypodiploid nuclei by flow cytometry. Data are the mean ± standard deviation of percentage of hypodiploid nuclei obtained in three different experiments, Graph in **A** shows all the experimental conditions, in **B** TcdB-treated EGCs and TcdB + CK-treated EGCs with and without inhibitors at 24 h, 48 h, and 72 h. Statistical analysis was performed by one-way ANOVA and Tukey’s multiple comparisons test. *P < 0.05, **P < 0.01, ***P < 0.001, ****P < 0.0001, ns *P* > 0.05

As reported (Fig. 1A) [9] flow cytometry analysis confirmed that among the EGCs treated with TcdB + CKs, the percentage of apoptotic cells approximately doubled compared to that among the cells treated with TcdB alone (Figs. 1A, 2A, B, 3A, 4A, B, 5A, 6A, B, 7A, B, 8A, 9A, B, 10, 11). Further, Western blot analyses showed that TcdB alone induced the activation of caspases-3 in EGCs (Figs. 2C, 3B-E, 4C, 5C, 6C, 7C, 8B, 9D), caspase-7 (Figs. 2D, 4D, 6D, 7D, 9E), and PARP (Figs. 2E, 4E, 6E, 7E, 9H), and these cleavage events increased approximately 50/100% after treatment with TcdB + CKs.

The pan-caspase inhibitor BAF completely inhibited apoptosis induced by both TcdB alone and TcdB + CKs (Fig. 2A, B) but had different effects on caspase activation by TcdB and TcdB + CKs. BAF reduced the cleavage of caspase-3 into a 17-kDa fragment by 64% after the administration of TcdB alone (Fig. 2C) but increased it by 29% after the administration of TcdB + CKs (Fig. 2C). The cleavage of caspase-7 into an active 20-kDa fragment was reduced by BAF by 85% after administration of TcdB alone (Fig. 2D) but was not significantly affected after administration of TcdB + CKs (Fig. 2D). Interestingly, BAF did not prevent the cleavage of PARP into the active 89-kDa fragment after administration of TcdB alone but rather increased it by 23% (Fig. 2E). In contrast, BAF reduced PARP cleavage by 26% after administration of TcdB + CKs (Fig. 2E).

These results suggest the involvement of other proteases in the transduction of pro-apoptotic signalling that seems in part different in TcdB and TcdB + CKs: (a) in TcdB-induced EGC apoptosis caspase-3 and caspase-7 are also cleaved by non-caspase proteases since BAF does not completely prevent their cleavage and activation. Furthermore, the cleavage/activation of PARP, a preferred caspase-3 substrate, to its 89-kDa form, was not inhibited by BAF further suggesting that non-caspase proteases concur also to PARP cleavage; and (b) also in TcdB + CK-induced EGC apoptosis caspase-3 and caspase-7 are mainly cleaved by non-caspase proteases because BAF increased the activation of caspase-3 and did not inhibit caspase-7 activation, but unlike TcdB alone, in TcdB + CK-induced apoptosis PARP cleavage was partially inhibited by BAF suggesting a minor involvement of other proteases in the effector phases of TcdB + CK-induced apoptosis. However, since BAF inhibited completely apoptosis there are two possible explanations: (1) the pro-apoptotic signalling triggered by TcdB alone and TcdB + CKs based on upstream non-caspase proteases in any case converges on executioner caspases or their substrates, (2) BAF has inhibitory effects on non-caspase proteases or in their apoptotic signalling.

Therefore, to understand whether the activity of BAF in our model has peculiar characteristics, we have used another well-known pan-caspase inhibitor Q-VD-OPh, a synthetic peptide that is potent, cell permeable, non toxic and irreversibly inhibits caspase activity, with several folds higher activity than the corresponding FMK caspase inhibitors [55–58], performing dose dependent experiments to evaluate the percentage of apoptotic cells (hypodiploid DNA content) by flow cytometry and the activation of caspase-3 by Western blot analysis in EGCs treated with TcdB or TcdB + CKs.

The pan-caspase inhibitor Q-VD-OPh at 50 μM, 25 μM, and 10 μM completely inhibited apoptosis induced by both TcdB alone and TcdB + CKs (Fig. 3A), at 2 μM reduced apoptosis of more than 83% in both conditions (Fig. 3A) and at 1 μM reduced apoptosis of about 60% in both conditions (Fig. 3A) while does not inhibit apoptosis at doses of 0.5 μM and below (Fig. 3A). Analysis of effect of Q-VD-OPh on caspase-3 activation demonstrates that Q-VD-OPh at 50 μM increased the cleavage of caspase-3 into a 17-kDa fragment by 287% after the administration of TcdB and by 140% after TcdB + CKs (Fig. 3B), Q-VD-OPh at 10 μM increased the cleavage of caspase-3 into a 17-kDa fragment by 47% after the administration of TcdB and by 21% after TcdB + CKs (Fig. 3C). Q-VD-OPh at 2 μM inhibited the cleavage of caspase-3 of about 35% after administration of TcdB alone (Fig. 3D) but increased the cleavage of caspase-3 of 23% after administration of TcdB + CKs (Fig. 3D), highlighting only at this dose a difference between TcdB and TcdB + CKs. Q-VD-OPh at 0.2 μM, which has no effect on apoptosis, does not affect the cleavage of caspase-3 into a 17-kDa fragment in both conditions (Fig. 3E). Therefore, Q-VD-OPh in the range of 50 μM and 10 μM prevented apoptosis but not the cleavage of caspase-3 in both conditions while at 2 μM, like to BAF, reduced apoptosis of more than 83% in both conditions but reduced caspase-3 cleavage only after administration of TcdB alone.

These results confirm that Q-VD-OPh such as BAF prevented execution of apoptosis but evidenced the activation of non-caspase proteases involved in the caspase-3 cleavage. It still remains to understand whether these non-caspases apoptotic signalling converge on the caspases and are then inhibited by the pan-caspase inhibitors, or if they are directly inhibited with a side effect of the inhibitors.

### Role of executioner caspase-3 and caspase-7

Another important point is to discriminate between induction and execution apoptotic signalling. To define the execution apoptotic signalling we evaluate the role of effector caspase-3 and caspase-7 in EGC apoptosis induced by TcdB and TcdB + CKs analysing the effects of Z-DEVD-fmk (a caspase-3 and caspase-7 inhibitor) [9, 17, 20, 21, 24, 28, 39, 59] on the percentage of apoptotic cells (hypodiploid DNA content) by flow cytometry, the activation of caspase-3 and caspase-7 (as measurement of upstream caspase activation) and PARP as measurement of executioner caspase activation, by Western blot analysis in EGCs treated with TcdB and TcdB + CKs.

The effector caspase inhibitor Z-DEVD-fmk at 2 μM reduced EGC apoptosis induced by TcdB by 27% but increased that induced by TcdB + CKs by 20% (Fig. 4A, B). Z-DEVD-fmk at 2 μM reduced the cleavage of caspase-3 to a 17-kDa fragment by 23% after administration of TcdB alone, but the cleavage of caspase-3 to the 17-kDa fragment was not affected by TcdB + CKs (Fig. 4C). Further, Z-DEVD-fmk at 2 μM reduced the cleavage of caspase-7 by more than 35% after administration of TcdB alone but as for caspase-3 cleavage did not affect caspase-7 cleavage after TcdB + CK administration (Fig. 4D). However, Z-DEVD-fmk at 2 μM reduced the cleavage of PARP by 48% after administration of TcdB alone and 28% after TcdB + CK administration (Fig. 4E). These results evidenced two important features: (1) executioner caspases are involved in about 30% of apoptosis induction, implying the involvement of a further pro-apoptotic signalling that does not pass through the effector caspases, (2) the pro-apoptotic signalling in TcdB + CKs is based more than in TcdB on apoptotic pathways not mediated by the effector caspases, caspase-3 and caspase-7.

However, to exclude that the concentration of Z-DEVD-fmk at 2 μM was too low to completely inhibit TcdB-triggered caspase activation in our model we performed concentration dependent experiments with Z-DEVD-fmk using the concentration of 20 μM and 10 μM, that were not toxic to EGCs as evidenced by analysis of cell viability by trypan blue assay, and evaluating the percentage of apoptotic cells (hypodiploid DNA content) by flow cytometry and on the activation of caspase-3 by Western blot analysis.

Also, higher concentrations of Z-DEVD-fmk consistently failed to rescue cells from TcdB-induced death (Fig. 5A). Z-DEVD-fmk both at 20 μM and 10 μM did not inhibit EGC apoptosis induced by TcdB and TcdB + CKs (Fig. 5A). Further Z-DEVD-fmk at 20 μM and 10 μM increased the cleavage of caspase-3 to a 17-kDa fragment, respectively, by 110% and by 60% after administration of TcdB alone (Fig. 5C), and by 17% after administration by TcdB + CKs (Fig. 5C).

These results further confirm that there is an important part of apoptosis which is independent of the effector caspases, caspase-3 and caspase-7, while they are cleaved and then activated in the presence of Z-DEVD-fmk which blocks the effector functions, then the apoptosis proceeds almost unchanged through a signalling that bypasses caspase-3 and caspase-7. Altogether these data that suggest a limited role of the executioner caspases in apoptosis leaving open the possibility that a role can be played by the initiator caspases through an effector pathway that does not pass through caspase-3 and caspase-7.

### Role of initiator caspase-8

To discriminate between induction and distal execution apoptotic signalling mediate by caspases in our model, because Tcds have been reported to activate caspase-8 in several cell models [17, 20–23], and TNF-α can activate apoptosis by the receptor-mediated pathway by recruiting and activating pro-caspase-8, which triggers the activation of apoptotic signalling also effector caspase independent [30, 31] [32, 33, 47–52, 63] we investigated the involvement/role of caspase-8 as initiator/upstream caspase and of the extrinsic pathway in EGC apoptosis induced by TcdB alone and TcdB + CKs. To this end, we analysed the effects of Z-IETD-fmk (a specific inhibitor of caspase-8) [17, 20, 21, 24] on the percentage of apoptotic cells (hypodiploid DNA content) by flow cytometry, the activation of caspase-3 and caspase-7 (as measurement of upstream caspase activation) and PARP (as measurement of executioner caspase activation) by Western blot analysis in EGCs treated with TcdB and TcdB + CKs.

Z-IETD-fmk reduced the apoptosis of EGCs induced by TcdB alone and that induced by TcdB + CKs by approximately 28% (Fig. 6A, B).

Western blot analysis demonstrated that Z-IETD-fmk did not affect the cleavage of caspase-3 into the 17-kDa fragment after administration of TcdB alone but reduced it by 24% after TcdB + CK administration (Fig. 6C). Z-IETD-fmk reduced the cleavage of caspase-7 into the active 20-kDa fragment by 30% after administration of TcdB alone but did not significantly affect it after TcdB + CK administration (Fig. 6D). Z-IETD-fmk reduced the cleavage of PARP into the 89-kDa fragment by more than 42% after administration of TcdB alone and after TcdB + CK administration (Fig. 6E).

These results indicate that caspase-8 is involved in caspase-7 activation but not caspase-3 activation in TcdB-induced apoptosis, while in TcdB + CK-induced apoptosis, the opposite phenomenon occurs, as caspase-8 is involved in caspase-3 but not caspase-7 activation, highlighting the contribution of caspase-8 to the part of apoptosis caspase-dependent in both situations through different signalling pathways. Moreover, because the reduction of PARP cleavage mediated by Z-IETD-fmk was greater than executioner caspase inhibition it seems that caspase-8 could lead to PARP cleavage independently of caspase-3 and caspase-7.

### Role of cathepsin B

Based on the above results suggesting that other proteases are involved in transduction of the apoptotic signal in EGC apoptosis induced by TcdB and TcdB + CKs particularly in executioner caspase cleavage and in effector phase of apoptosis, we investigated the involvement of cathepsin B by analysing the effect of CA-074Me (a cathepsin B inhibitor) [39, 43] on apoptosis through evaluation of the percentage of apoptotic cells (hypodiploid DNA content) by flow cytometry, and caspase-3, caspase-7 (as measurement of upstream caspase and non-caspase activation) and PARP (as measurement of executioner caspase or non caspase-mediated cleavage) by Western blot analysis in EGCs treated with TcdB and TcdB + CKs.

CA-074Me 10 μM reduced the apoptosis of EGCs by approximately 35% upon administration of both TcdB alone and TcdB + CKs (Fig. 7A, B). However, the reduction in apoptosis was not accompanied by the inhibition of effector caspases but rather paradoxically by an increase in their activation (Fig. 7C, D). 10 μM CA-074Me strongly increased the cleavage of caspase-3 into the 17-kDa products by 352% after administration of TcdB alone (Fig. 7C) and 210% after TcdB + CK administration (Fig. 7C). CA-074Me also increased the cleavage of caspase-7 by 210% after administration of TcdB alone (Fig. 7D) and 64% after TcdB + CK administration (Fig. 7D). CA-074Me increased the cleavage of PARP by 33% after administration of TcdB alone (Fig. 7E) and by 55% after TcdB + CKs (Fig. 7E).

However, the cathepsin B inhibitor (CA-074Me) did not reduced the cytopathic effects induced by TcdB and TcdB + CKs (data not shown).

Successively, because we found that 10 μM CA-074Me itself induces caspase activation, we have titrated down CA-074Me using the dose of 5 μM and 1 μM on analysis of apoptosis (hypodiploid DNA content) by flow cytometry and caspase-3 activation by Western blot.

CA-074Me at 5 μM reduced the apoptosis of EGCs by approximately 26% in both conditions (Fig. 8A) while at 1 μM does not significantly inhibited apoptosis in both conditions (Fig. 8A). Regarding caspase-3 activation, CA-074Me at 5 μM by itself (Fig. 8B middle) and upon administration of both TcdB alone and TcdB + CKs (Fig. 8B middle) increased the cleavage of caspase-3 into a 17-kDa fragment (Fig. 8B middle), in contrast at 1 μM CA-074Me did not increase caspase-3 activation after TcdB alone (Fig. 8B right) and still increased caspase-3 cleavage into 17-kDa fragment by itself and after TcdB + CKs (Fig. 8B right) but did not inhibit TcdB and TcdB + CK induced apoptosis (Fig. 8A). Then, a lower dose of CA-074Me cannot use to inhibit cathepsin B in our model.

These results demonstrate that cathepsin B plays a role in TcdB-induced EGC apoptosis and induction of the apoptotic pathway is caspase-3- and caspase-7-independent because the inhibition of apoptosis was accompanied by an increase in the activation of both caspases. Correlation of the strong increase in caspase-3 and caspase-7 cleavage with the inhibition of cathepsin B seems to suggest a role for cathepsin B in regulating the mechanisms of caspases cleavage suggesting a reciprocal regulation between these two families of proteases. Cathepsin B could inhibit caspase-8, and inhibiting cathepsin B, caspase-8 could be reactivated and favours so the increase of cleavage of effector caspases. Further, cathepsin B could also amplify the apoptotic signal by degrading XIAP [35–41], or it is possible that cathepsin B controls the levels of active fragments of the effector caspases. Therefore, this could explain how the pan-caspase inhibitors used, BAF and Q-VD-OPh, inhibiting also the cathepsins, would favour the increase of cleavages by other proteases non-caspases such as calpains.

### Role of calpains and Ca^2+^

Calpains, non-lysosomal cysteine proteases activated by an increase in intracellular Ca^2+^, are found in all mammalian cells and mediate apoptosis in a caspase-dependent or caspase-independent manner [35–40, 60–62]. Since it has been reported that TcdB induces an increase in Ca^2+^ in intoxicated intestinal cells [66, 67] and it has been suggested that TcdA activates non-caspase proteases during intestinal cell apoptosis [22], TcdB and especially TcdB + CKs may induce an increase in Ca^2+^ in EGCs that could activate calpains. Thus, the involvement of calpains in TcdB- and TcdB + CK-induced EGC apoptosis particularly in executioner caspase cleavage and in effector phase of apoptosis was studied by analysing the effect of PD150606 (a specific inhibitor of calpains that targets the Ca^2+^-binding site) [39, 60–62] on the percentage of apoptotic cells (hypodiploid DNA content), as measured by flow cytometry, on the caspase-3 and caspase-7 (as measurement of upstream caspase and non-caspase activation) and PARP (as measurement of executioner caspase or non caspase-mediated cleavage) by Western blot analysis, in EGCs treated with TcdB and TcdB + CKs**.**

The results of flow cytometry analysis showed that PD150606 strongly inhibited apoptosis by approximately 75% upon administration of TcdB alone (Fig. 9A, B) and 62% upon TcdB + CK administration (Fig. 9A, B), suggesting a strong involvement of calpains.

To confirm calpain activation, we evaluated the effect of PD150606 on the cleavage of α-spectrin [39, 60–62, 70, 71], a cytoskeletal protein, into two fragments, a 145-kDa cleavage fragment characteristic of the activation of calpains and a 120-kDa cleavage fragment characteristic of caspase activation, by Western blot analysis.

Either TcdB alone or TcdB + CKs activated calpains during EGC apoptosis, and the level of activation was similar under both conditions (Fig. 9C). Indeed, the 145-kDa fragment was strongly detected at 24 h under both conditions (Fig. 9C), and PD150606 reduced its formation by 25% under both conditions (Fig. 9C).

Furthermore, assessment of α-spectrin cleavage confirmed caspase activation, since the 120-kDa fragment of α-spectrin was generated under both conditions (Fig. 9C) and PD150606 reduced its formation by 34% after treatment with TcdB alone (Fig. 9C) and 21% after TcdB + CK treatment (Fig. 9C). Taken together, these data indicate that TcdB alone and TcdB + CKs activated calpains and confirm caspase activation, suggesting that calpains may contribute to the activation of caspases. Indeed, PD150606-mediated inhibition of calpains also reduced the generation of 120-kDa fragments by caspase activation.

We found also that PD150606 inhibited the cleavage of caspase-3 by 73% and of caspase-7 by 86% after treatment with TcdB alone (Fig. 9D, E) and inhibited the cleavage of caspase-3 and caspase-7 by approximately 25% after TcdB + CK treatment (Fig. 9D, E). Further, PD150606 inhibited the cleavage of PARP by approximately 24% under both conditions (Fig. 9F).

These results demonstrate that calpain activation contributed strongly to apoptosis upon treatment with both TcdB alone and TcdB + CKs and that the apoptotic signalling is mediated by the activation of caspase-3 and caspase-7 but also by caspase-independent pathways likely mediated by direct cleavage of PARP. However, upon treatment with TcdB + CKs, while the rate at which apoptosis was inhibited was similar to that upon treatment with TcdB alone, the role of calpains in the cleavage of caspase-3 and caspase-7 seems less.

Since calpains are Ca^2+^-dependent proteases and both Tcds and TNF-α can induce Ca^2+^ influx [18, 19, 24, 65–67], which could be responsible for calpain activation [36–40], to define the effect of Ca^2+^ on the apoptotic activity of TcdB and source of Ca^2+^, we analysed (a) the role of extracellular Ca^2+^ influx using both the extracellular Ca^2+^ chelator EGTA and the intracellular Ca^2+^ chelator BAPTA-AM, and (b) the role of Ca^2+^ channels using two different types of Ca^2+^ channel blockers, nifedipine [65, 66] and NiCl_2_ [65].

We found that it was impossible to analyse the role of Ca^2+^ influx on the apoptotic activity of TcdB using EGTA and BAPTA-AM because even when the chelators were used at very low concentrations, EGCs detached from the cell culture plate and stopped growing, preventing treatment with TcdB.

To analyse the effect of the Ca^2+^ channel blockers, EGCs were treated with NiCl_2_ (a specific inhibitor of low-voltage-activated T-type calcium channels) [65] or nifedipine (a specific inhibitor of L-type voltage-dependent Ca^2+^ channels) [65, 66] as described in the Materials and methods section.

NiCl_2_ (20 μM) and nifedipine (1 μM) did not affect EGC apoptosis after treatment with TcdB alone (data not shown) or TcdB + CKs (data not shown), suggesting that activation of calpains is not mediated by an increase in intracellular Ca^2+^ due to the activation of L-type and T-type Ca^2+^ channels.

To better understand the possible interactions between the activated caspase and cathepsin pathways in our model, we evaluated the effects of a pan-caspase inhibitor (BAF) and a cathepsin B inhibitor (CA-074Me) on α-spectrin cleavage into the 145-kDa and 120-kDa fragments. The results obtained showed that BAF increased by approximately 30% the 145-kDa α-spectrin fragment under both conditions (Fig. 9G) but reduced the 120-kDa α-spectrin fragment by 24% after TcdB alone (Fig. 9G) and 45% after TcdB + CK treatment (Fig. 9G), suggesting that caspases are not involved in calpain activation but rather that caspase activation is mediated by calpain activation. Furthermore, the demonstration that inhibition of caspases with BAF increased calpain activation suggest that there is a complex interplay/regulation between the activity of different families of proteases where when a protease family is inhibited, the other families are no longer under control and can thus acquire a greater activity. CA-074Me reduced only the cleavage of α-spectrin into the 120-kDa fragment by approximately 25% under both conditions (Fig. 9H), indicating that cathepsin B mediated caspase activation but are not involved in calpain activation.

### Role of the mitochondria-mediated pathway in EGC apoptosis induction by TcdB and TcdB + CKs

Previously, we demonstrated that TcdB-induced EGC apoptosis is caspase-dependent and mitochondria-independent [9], but TNF-α, by activating caspase-8 [30–33], cathepsins [42–44] and calpains [36–40], can cleave/activate Bid, which can induce apoptosis through the mitochondrial pathway. Therefore, we investigated the involvement of the mitochondrial pathway by analysing the effects of caspase, calpain and cathepsin inhibitors on caspase-9 and Bid activation by Western blot analysis in EGCs treated with TcdB and TcdB + CKs.

TcdB alone induced the activation of caspase-9 in EGCs to produce the 40/38-kDa fragments, and this cleavage was strongly increased by approximatively 130% after treatment with TcdB + CKs (Fig. S1A-E). Regarding Bid, we did not find cleavage/activation under either condition, but we found its expression to be increased 53% after treatment with TcdB + CKs rather than TcdB alone (Fig. S1A-E).

BAF, Z-DEVD-fmk, and Z-IETD-fmk did not prevent the cleavage of caspase-9 into the 40/38-kDa fragments but significantly increased its cleavage after treatment with TcdB alone by 64%, 44% and 64%, respectively (Fig. S1A-C), while caspase-9 cleavage was reduced by 28%, 53%, and 28% after TcdB + CK treatment, respectively (Fig. S1A-C). PD150606 did not significantly affect caspase-9 cleavage under both condition (Fig. S1D), but CA-074Me significantly increased the cleavage of caspase-9 by approximately 100% after treatment with TcdB alone and by approximately 50% after TcdB + CK treatment (Fig. S1E).

Analysis of Bid expression after inhibitor treatment showed that BAF (Fig. S1A), Z-DEVD-fmk (Fig. S1B), Z-IETD-fmk (Fig. S1C) and PD150606 (Fig. S1D) did not significantly change Bid expression after treatment with TcdB alone (Fig. S1A-D) but, Z-DEVD-fmk (Fig. S1B), Z-IETD-fmk (Fig. S1C) and PD150606 (Fig. S1D) reduced Bid expression by 27% (Fig. S1B), 34% (Fig. S1C) and 18% (Fig. S1D) after TcdB + CK treatment, respectively. On the contrary, CA-074Me increased Bid expression by 18% after treatment with TcdB alone (Fig. S1E) but reduced it by 13% after TcdB + CK treatment (Fig. S1E).

Altogether, these data indicate that caspase-9 and Bid were involved in TcdB + CK-induced apoptosis but not in TcdB-induced apoptosis.

### Effect of the combination of Z-DEVD-fmk with PD150606 or CA-074Me in EGC apoptosis induced by TcdB and by TcdB + CKs

Since the results obtained using the pan-caspase inhibitors and the inhibitor of effector caspases (Z-DEVD-fmk) suggest the involvement of other apoptotic signalling pathways, we examined the effect of the combination of Z-DEVD-fmk (2 μM) with PD150606 (100 μM) or CA-074Me (10 μM) to try to obtain complete apoptosis inhibition as obtained with BAF, evaluating the percentage of apoptotic cells (hypodiploid DNA content) by flow cytometry.

The results showed that neither the combination of Z-DEVD-fmk with PD150606 or Z-DEVD-fmk with CA-074Me had a synergistic effect on apoptosis inhibition under both conditions (Fig. 10). We found that Z-DEVD-fmk with PD150606 inhibited apoptosis by approximately 54% upon administration of TcdB alone (Fig. 10) and 46% upon TcdB + CK administration (Fig. 10) and Z-DEVD-fmk with CA-074Me inhibited apoptosis by approximately 44% upon administration of TcdB alone (Fig. 10) and 33% upon TcdB + CK administration (Fig. 10).

These results suggest that the inhibition of the effector caspases with Z-DEVD-fmk in association with cathepsin B or calpain inhibitor fails to block all the three families of proteases involved in apoptosis induced by TcdB or TcdB + CKs, indicating that treatment with each combination leaves free an apoptotic pathway that continues to induce apoptosis.

### Kinetics of EGC apoptosis induced by TcdB and by TcdB + CKs and effect of the inhibitors

Since the cell death events could be shifted temporally in the presence of inhibitors, we have addressed this issue measuring apoptosis under inhibitor conditions at longer timepoints extending the period of intoxication up to 72 h. To this end, we have analysed the effect of pan-caspase inhibitors BAF (50 μM) and Q-VD-OPh (2 μM), calpain inhibitor PD150606 (100 μM), and cathepsin B inhibitor CA-074Me (10 μM), on apoptosis of EGCs induced by TcdB alone and TcdB + CKs evaluating the percentage of apoptotic cells (hypodiploid DNA content) by flow cytometry at 24 h, 48 h and 72 h in EGCs treated with TcdB and TcdB + CKs to increase the dynamic range of the apoptosis assay to clarify the interpretation of our results.

Flow cytometry analysis demonstrated that regarding apoptosis induced by TcdB alone at 48 h, the percentage of apoptosis did not significantly change with respect to that of 24 h, while at 72 h it increased by 65% (Fig. 11). We found 11.3% at 24 h, 12.8% at 48 h and 18.7% at 72 h (Fig. 11). Using the pan-caspase BAF and Q-VD-OPh inhibitors, the calpain (PD150606) and cathepsin B (CA-074Me) inhibitors, the percentage of apoptotic cells present at 24 h remained approximately constant at 48 h and slowly increased at 72 h remaining anyway strongly inhibited at every time examined (Fig. 11). This confirms that already at 24 h the majority of cells susceptible to the apoptotic activity of TcdB at the concentration of inhibitor used were involved, so there are no cell death events that the inhibitor could have been delayed in the first 24 h and manifest later.

On the other hand, regarding apoptosis induced by TcdB + CKs, the percentage of apoptosis increased progressively up to 72 h increasing by 100% at 48 h and by 150% at 72 h (Fig. 11). We found about 24.3% apoptosis at 24 h, about 48.8% at 48 h and about 60.6% at 72 h (Fig. 11). These results highlight a progressive increase in apoptosis also in cells which after 24 h are not yet apoptotic. However, in presence of the pan-caspase inhibitors BAF and Q-VD-OPh, of calpain (PD150606) and cathepsin B (CA-074Me) inhibitors, the percentage of apoptotic cells present at 24 h progressively increased at 48 h and 72 h (Fig. 11) but a part of apoptosis remains nevertheless inhibited even in the following days because the value of apoptosis was always strongly lowered compared to the value of apoptosis observed at 48 h and 72 h in EGCs treated with TcdB + CKs without inhibitors (Fig. 11), indicating that the inhibitor is able to block the apoptotic signalling in one part of the cells programmed for apoptosis which has not manifested apoptosis within the first 24 h.

The results are very suggestive because they demonstrate that apoptosis in the cells treated with TcdB + CKs progressively increased, and that the inhibitors can block a portion of cells that are still not apoptotic at 24 h, but which have already activated an apoptotic program where the inhibitors block all the cells that have started the apoptotic process, thus indirectly confirm the difference of apoptotic signals activated by TcdB and by TcdB + CKs.

### Role of anti-apoptotic signalling in the apoptosis of EGCs induced by TcdB and by TcdB plus TNF-α and IFN-γ (CKs)

#### Role of the NF-κB pathway

The binding of TNF-α to its receptor results in the degradation of the IκBα repressor and release of the transcription factor NF-κB, which migrates into the nucleus and induces the expression of anti-apoptotic genes [30–33, 47]. Therefore, we analysed the possible involvement of NF-κB by evaluating the change in IκBα expression by Western blot analysis in EGCs treated as described in the Materials and methods section.

The expression of IκBα increased by approximately 50% after treatment with TcdB alone with respect to the control (Fig. S2) but decreased by approximately 33% after TcdB + CK treatment with respect to TcdB alone (Fig. S2), suggesting that TcdB inactivated NF-κB signalling, whereas TcdB + CKs restarted it.

#### Role of AKT activation

The AKT signalling pathway plays a major role in cellular survival in various cell types [27, 50, 51, 63], and AKT activation can be modulated by TNF-α and IFN-γ [32, 52]. Since we previously demonstrated that TcdB at 10 ng/ml induced the activation of AKT in EGCs [53], we evaluated whether AKT was activated by 0.1 ng/ml TcdB alone or by TcdB + CKs and ultimately whether AKT was involved in EGC apoptosis under both conditions. First, we evaluated AKT phosphorylation by Western blot analysis and then evaluated the effect of the AKT inhibitor perifosine [53, 63, 64] on AKT phosphorylation (Western blotting) and apoptosis (hypodiploid DNA content; flow cytometry).

AKT phosphorylation was increased by approximately 40% after treatment with TcdB with respect to the control (Fig. S3A), 200% after treatment with TcdB + CKs with respect to the control and 120% after treatment with TcdB + CKs with respect to TcdB alone (Fig. S3A).

AKT inhibitor perifosine inhibited AKT phosphorylation by approximately 45% after treatment with TcdB alone (Fig. S3B) and 38% after treatment with TcdB + CKs (Fig. S3B). Perifosine reduced the percentage of apoptotic cells after TcdB treatment by approximately 34% (Fig. S3C and D) but did not change the percentage of apoptotic cells after TcdB + CK treatment (Fig. S3C and D), suggesting that AKT phosphorylation plays a different role in the effects of TcdB and TcdB + CKs, with the role in TcdB-induced apoptosis likely pro-apoptotic.

To better define the role of AKT phosphorylation, we also evaluated caspase-3 activation in EGCs treated with perifosine. Surprisingly, inhibition of AKT phosphorylation strongly increased caspase-3 activation under both conditions, as shown by a 74% increase in the active 17-kDa fragment after treatment with TcdB alone (Fig. S3E) and 94% after treatment with TcdB + CKs (Fig. S3E), suggesting an inverse correlation between AKT and caspase activation and highlighting the complexity of these signalling pathways upon administration of TcdB and TcdB + CKs.

Subsequently, we evaluated whether AKT phosphorylation was dependent on the activation of apoptotic signalling by evaluating the effect of caspase, calpain and cathepsin B inhibitors on AKT phosphorylation.

BAF did not change AKT phosphorylation after treatment with TcdB alone (Fig. S4A) but increased AKT phosphorylation by 21% after TcdB + CK treatment (Fig. S4A). Z-DEVD-fmk and Z-IETD-fmk increased AKT phosphorylation by 24% (Fig. S4B) and 52% (Fig. S4C), respectively, after treatment with TcdB alone (Fig. S4B and C) and by 46% (Fig. S4B) and 78% (Fig. S4C), respectively, after TcdB + CK treatment (Fig. S4B and C). PD150606 increased AKT phosphorylation by 30% after treatment with TcdB alone (Fig. S4D) but did not change AKT phosphorylation after TcdB + CK treatment (Fig. S4D). CA-074Me reduced AKT phosphorylation by 34% after treatment with TcdB alone (Fig. S4E) but did not change AKT phosphorylation after TcdB + CK treatment (Fig. S4E).

Altogether, these results confirm the strong involvement of AKT activation in our system, indicate that AKT phosphorylation is caspase and calpain activation-independent but cathepsin B activation-dependent, and suggest an inverse correlation between caspase activation and AKT phosphorylation. Furthermore, these results highlight the peculiarity of each activated apoptotic pathway and the interactions between the activated apoptotic signalling pathways, but a more complete understanding would require further study. Perifosine indicates that AKT phosphorylation exerts a pro-apoptotic effect, particularly after TcdB treatment, but the caspase, calpain, and cathepsin inhibitors suggest that AKT activation play an anti-apoptotic/pro-survival role.

#### Role of JNK activation

Since we previously demonstrated that 0.5, 5 and 10 ng/ml TcdB induced the activation of JNK in EGCs [10, 53] and that the JNK inhibitor SP600125 significantly attenuated TcdB-induced DNA fragmentation and contributed to the survival of EGCs [10, 53], we analysed its role in apoptosis induced by TcdB at 0.1 ng/ml and by TcdB + CKs by first evaluating the activation of JNK by Western blot analysis and then assessing the effect of the JNK inhibitor SP600125 on JNK phosphorylation (Western blotting) and apoptosis (hypodiploid DNA content; flow cytometry) [10, 53].

Under our experimental conditions, JNK phosphorylation was not increased by TcdB at 0.1 ng/ml alone or by TcdB + CKs (Fig. S5A), suggesting that JNK is not involved in TcdB- or TcdB + CK-induced apoptosis. Furthermore, the JNK inhibitor SP600125 did not inhibit JNK phosphorylation but rather tended to increase it (Fig. S5B). However, the JNK inhibitor SP600125 increased apoptosis (Fig. S5C and D). SP600125 increased the percentage of apoptotic cells by approximately 80% upon TcdB treatment (Fig. S5C and D) and by approximately 27% upon TcdB + CK treatment (Fig. S5C and D). Finally, SP600125 strongly increased caspase-3 activation under every condition examined, as shown by the increased production of the active 17-kDa fragment (Fig. S5E).

These preliminary results suggest that JNK promotes survival in TcdB- and TcdB + CK-mediated apoptosis through a mechanism that requires further in-depth study.

## Discussion

Previously, we demonstrated that TcdB induces apoptosis in EGCs and that TcdB plus TNF-α and IFN-γ (TcdB + CKs) increase apoptosis by enhancing the cytotoxic activity of TcdB [9]. We also demonstrated that TcdB-induced EGC apoptosis is caspase-dependent and mitochondria-independent and that stimulation with CKs enhances susceptibility to TcdB-induced apoptosis by increasing caspase-3, caspase-7, caspase-9 and PARP activation [9]. Our previous results suggested caspase-dependent apoptotic signalling under both conditions, but the possible roles of other apoptotic pathways were not evaluated. It is possible that apoptotic signalling induced by TcdB in EGCs is more complex than we previously described because Nottrott et al. suggested that TcdA-induced apoptosis in HT-29 cells depended on the activation of both caspase-3 and non-caspase proteases, particularly cathepsins [22]. Furthermore, the enhancement of TcdB cytotoxicity in EGCs by CKs can induce apoptosis by the extrinsic pathway, which is triggered by not only caspase-8 activation but also cathepsin B activation. Furthermore, cathepsin B destabilizing the lysosomal membrane can contribute to this signal to increase apoptosis or alter the balance between the pro-apoptotic (cysteine proteases, JNK and ROS) and pro-survival (NF-κB or PI3K/AKT) arms of the TNF-α signalling pathways [30–33, 47–52, 63]. Moreover, both TcdB and TNF-α, in different ways, can alter Ca^2+^ influx and modify even if, for short periods, intracellular Ca^2+^ levels which can activate calpains and thus permit calpain-mediated apoptotic signalling [35–40, 65, 66].

A further open question was whether TcdB + CKs would enhance the apoptotic activity of TcdB, although CKs alone are not cytotoxic, or whether TcdB, by blocking Rac1 [4–8], would prevent CKs from activating the anti-apoptotic pathway, which is essential for TNF-α to carry out its physiological activity [30–33].

In an attempt to address these issues, we analysed apoptotic signalling pathways activated by TcdB alone and by TcdB + CKs in EGCs.

**Fig. 12:**
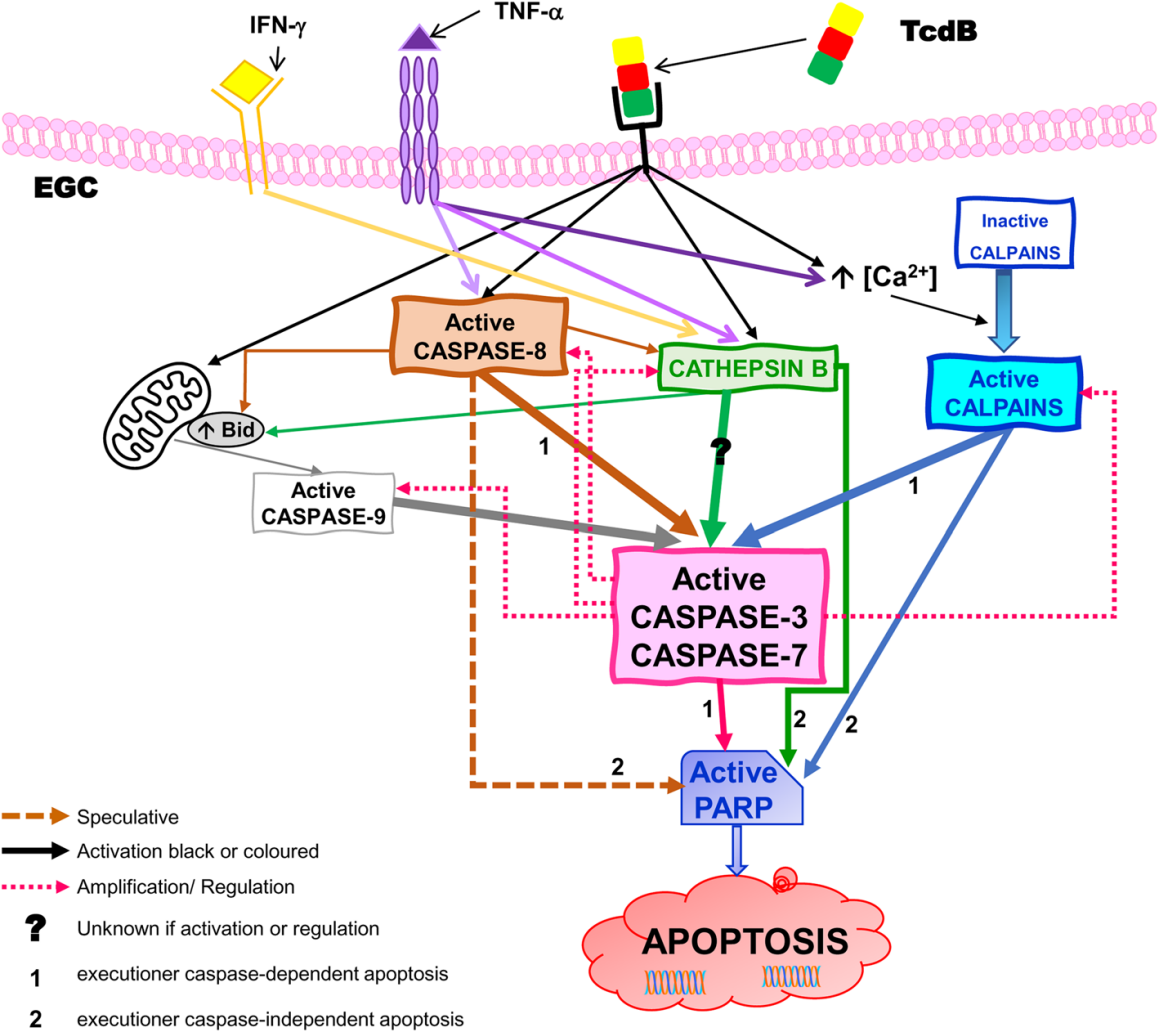
Schematic diagram showing the proposed apoptotic pathways activated by TcdB and TcdB + CKs. Schematic of the possible signalling pathways and of their interplay involving caspases, calpains and cathepsin B in the induction and execution of apoptosis induced by TcdB and enriched by TNF-α and IFN-γ (TcdB + CKs). TcdB *C. difficile* toxin B, EGC enteric glial cell

In this study, we demonstrate for the first time that TcdB alone and TcdB + CKs induced apoptosis in EGCs by activating three protease families (Fig. 12) caspases, cathepsins and calpains, which all are involved both in induction and execution apoptotic signalling but to different degrees in TcdB and TcdB + CKs especially as regards to apoptotic signal transduction towards downstream effects (apoptosis) (Fig. 12).

Our results show that Z-DEVD-fmk at 2 μM inhibited caspase-3, caspase-7 and apoptosis only upon treatment with TcdB (by 27%), indeed, we found a moderate inhibition of caspase-3 and a strong reduction in caspase-7 and PARP activation suggesting a more important role of caspase-7 in apoptosis. These data also demonstrate that inhibiting effectors caspase-3 and caspase-7 with Z-DEVD-fmk about 70% apoptosis was not inhibited, suggesting that can be involved caspases that do not go through executioner caspases to induce apoptosis but also other proteases such as cathepsins and calpains that could mediate apoptotic signal pathways in a caspase-3 and caspase-7 independent manner likely through direct PARP cleavage [35]. Moreover, the observation that Z-DEVD-fmk did not affect caspase-9 cleavage or the increase in Bid expression upon treatment with TcdB alone confirmed that caspase-3 and caspase-7 activation upon TcdB treatment did not activate the mitochondrial apoptotic pathway. On the other hand, instead of inhibiting apoptosis, under TcdB + CK conditions, apparently unexpectedly Z-DEVD-fmk treatment increased apoptosis, and consistent with these data, we did not find caspase-3 or caspase-7 to be inhibited suggesting that in TcdB + CKs apoptotic signalling does not pass through caspase-3 and caspase-7 and likely the execution of apoptosis could be mediated mainly by non-caspase proteases through direct PARP cleavage as reported in several apoptotic models [35]. However, in TcdB + CKs, caspase-9 cleavage and Bid expression were reduced. This suggests that activation of caspase-9 and increase in Bid expression in TcdB + CKs is facilitated by apical initiator caspases and by an amplification loop mediated by effectors caspases. Therefore, Z-DEVD-fmk inhibits only a portion of the apoptotic signal that passes through the caspase-3 and caspase-7 in TcdB while in TcdB + CKs it increased apoptosis. These data are strongly suggestive for an involvement of caspase-independent apoptotic signals. Furthermore, the limited ability of Z-DEVD-fmk to inhibit apoptosis is not due to the dose of the inhibitor used because higher doses of Z-DEVD-fmk do not inhibit apoptosis even after TcdB alone and with the unexpected result that caspase-3 activation strongly increases under both conditions. This is further evidence that there is caspase-independent apoptosis which is more evident when are strongly inhibited the executioner caspases.

The possible involvement of other signalling pathways is also suggested by the results obtained with BAF, a pan-caspase inhibitor, which almost completely inhibited apoptosis upon treatment with TcdB or TcdB + CKs. Upon TcdB treatment, this inhibition was accompanied by a strong reduction in caspase-3 and caspase-7 cleavage without an effect on Bid expression, and caspase-9 and PARP cleavage was rather increased, whereas upon TcdB + CK treatment, BAF-mediated inhibition of apoptosis was accompanied by an increase in caspase-3 activation, no significant change in Bid expression or caspase-7 activation, and a significant reduction in caspase-9 and PARP activation. The results on the effects of BAF on caspase-3 cleavage and Bid expression under TcdB + CK conditions are in agreement with the results of Nottrott et al., who demonstrated that the pan-caspase inhibitor Z-VAD-fmk, although prevent TcdA-induced HT-29 cell apoptosis, did not reduce Bid expression or caspase-3 cleavage in TcdA-induced HT-29 cell apoptosis, suggesting that a non-caspase protease is involved in their activation [22]. However, unlike Nottrott et al. [22], we found that when apoptosis was induced by TcdB alone, the majority of caspase-3 and caspase-7 activation was caspase-dependent because it was strongly inhibited by the pan-caspase inhibitor BAF while caspase-9 activation was caspase-independent because it was increased by BAF. On the contrary, when apoptosis was induced by TcdB + CKs, the majority of caspase-3 and caspase-7 activation was caspase-independent because was not inhibited by BAF while caspase-9 and PARP activation was partially caspase-dependent because it was partially inhibited by BAF. Although these effects of BAF seem surprising, they are not exclusive of BAF, because the same results were also found using the pan-caspase inhibitor Q-VD-OPh. Q-VD-OPh at 2 μM strongly inhibited apoptosis (about 84%) induced by TcdB and TcdB + CKs, and inhibited caspase-3 activation in TcdB but not in TcdB + CKs. Moreover, increasing the concentration of Q-VD-OPh at 50 and 10 μM we found completely inhibition of apoptosis but strong increase in caspase-3 cleavage in both conditions.

Then overall, our data showing that TcdB and TcdB + CKs activated caspase-3 and caspase-7, but specific executioner caspase inhibitor (Z-DEVD-fmk) at 2 μM blocks only by about 30% apoptosis in TcdB but did not block apoptosis in TcdB + CKs and further does not inhibit apoptosis at higher concentrations in both conditions; while pan-caspase inhibitors completely inhibited apoptosis but not the caspase-3 and caspase-7 cleavage that so could be mediated by non-caspase proteases, suggest: (a) that TcdB and TcdB + CKs induces a partially caspase-dependent but also caspase-independent proximal apoptotic signalling that induces activation of effectors caspase-3/caspase-7, and (b) the presence of a partially caspase-dependent, above all in apoptosis induced by TcdB alone, and strong caspase-independent distal apoptotic signalling under both conditions, in agreement with what has been described by other authors regarding the induction of apoptosis by TcdA in HT-29 and OVCAR3 cells [21, 22]. The pan-caspase inhibitors BAF and Z-VAD-fmk were shown that they can inhibit cathepsins [72], and Nottrott et al., using Z-VAD-fmk, demonstrated that TcdA-induced apoptosis in HT-29 cells was caspase-3 dependent but caspase activation was inhibited by the calpain/cathepsin inhibitor ALLM [22]. Thus, it is possible that even in our model while BAF inhibited caspase-3-mediated signalling, particularly when apoptosis was induced by TcdB + CKs, BAF can also inhibit other proteases, such as cathepsins, which in our model could be activated by lysosomal destabilization and/or by apoptotic signalling triggered by TNF-α and IFN-γ.

However, since Q-VD-OPh gave the same results obtained using BAF, and there is no evidence that Q-VD-OPh can inhibit cathepsin B or other protases [55–58] we might hypothesize that executioner caspase-3 and caspase-7 are cleaved by different types of upstream proteases that are not inhibited by BAF as calpains and cathepsin B, which could be more strongly activated after caspase-inhibition with BAF for the complex interplay and crosstalk due to the activation and reciprocal regulation of different families of proteases between themselves [70] as we will see later in the discussion. This can be also potentiated and differentially regulated in TcdB + CKs by the contemporaneity of activation of CK dependent signalling.

Therefore, we postulate a model of apoptosis induced by TcdB and TcdB + CKs with a caspase-dependent and caspase-independent induction apoptotic signalling (Fig. 12), because activation of executioner caspase-3 and caspase-7 inhibited by BAF could be mediated by initiator caspases (caspase-8 and caspase-9) [30, 31, 58] but the remaining activation that was not inhibited by BAF could be mediated by caspase-independent apoptotic signalling, refereed to calpains and cathepsins [36–40, 42, 43], that in turn are mutually regulated by a complex interplay [30, 31, 36–40, 42, 43, 58] contributing differently to apoptosis pathways in TcdB and TcdB + CKs. An important clue about the presence of a caspase-independent execution apoptotic signalling comes from the results with Z-DEVD-fmk that reduced apoptosis only by about 30% in TcdB but not in TcdB + CKs.

A specific inhibitor of cathepsin B, CA-074Me, inhibited apoptosis upon treatment with TcdB and TcdB + CKs by 35%, accompanied by a strong increase in the pro-apoptotic cleavage of caspase-3 (17-kDa) and caspase-7 and a further increase in PARP activation. Therefore, cathepsin B activation is involved in the apoptosis of EGCs induced by TcdB and TcdB + CKs but because the reduction in apoptosis induced by CA-074Me was not based on effector caspase inhibition they acted by a caspase-independent effector pathway. Further, most importantly these data also suggest that cathepsin B could have a regulatory role in degradation of active fragments of caspase-3 and caspase-7 since inhibition of cathepsin B causes a strong increase in the caspase-3/caspase-7 fragments. It is therefore possible that some caspases may in turn regulate the activation or activity of cathepsin B needed to regulate the levels of active fragments of the effector caspases. Thus, BAF, by inhibiting the caspases leads to unlocking of the activation/activity of cathepsin B which checks/regulates the levels of active caspase-3 and caspase-7 fragments. However, these active fragments are unable to induce signal transduction towards downstream effects and then apoptosis due to the inhibitory presence of BAF. It is possible that upon treatment with TcdB alone, the activation of cathepsins B is a consequence of the inhibition of Rac1, which plays a role in lysosomal stabilization [38, 42–46, 68, 69] and therefore in the release of pro-apoptotic cathepsin B, while upon treatment with TcdB + CKs, the inhibition of Rac1 by TcdB and TNF-α has an additive effect on the cell that, through the activation of NF-κB, induces an anti-apoptotic response to antagonize the apoptotic arm [30–33]. However, under these conditions, this anti-apoptotic response cannot be activated due to the blockade of Rac1 by TcdB, and pro-apoptotic signalling goes on through lysosomal destabilization and the consequent activation of cathepsin B.

The role of Ca^2+^ is central in the regulation of cellular homeostasis, and even transient Ca^2+^ fluctuations can induce apoptosis [36–40, 65, 66, 70, 71]. Since TcdB induces Ca^2+^ signalling [18, 19, 67] and calpains, a family of proteases that are very sensitive to variations in intracellular Ca^2+^ levels, can activate apoptotic signalling and because in different models of apoptosis, they seem to constitute the earliest signal for apoptosis [36–40, 70, 71], we analysed calpain activation and the possible role of calpains using the specific inhibitor PD150606.

Our results demonstrate for the first time that TcdB activated calpains in EGCs and that calpains are involved in apoptosis through a caspase-dependent and caspase-independent pathway. Upon TcdB alone we found that: (a) α-spectrin was cleaved into a 145-kDa fragment, specific for calpain protease activity, (b) PD150606 inhibited the cleavage of α-spectrin into the 145-kDa fragment, and (c) TcdB-induced apoptosis was greatly reduced (by approximately 75%), accompanied by a very strong, concomitant inhibitory effect on caspase-3 (by approximately 73%) and caspase-7 (by approximately 86%) activation and a significant decrease in PARP activation (by approximately 24%). PD150606 did not affect caspase-9 activation or Bid expression, highlighting how calpains in TcdB activate apoptotic execution signalling in a mitochondrial-independent but caspase-3- and caspase-7-dependent manner and suggesting that residual apoptosis that is not inhibited by PD150606 is not mediated by effector caspases and may be due to the activity of cathepsin B (approximately 25/30%). Regarding the effect of PD150606 upon TcdB + CK treatment, we found that calpains were also activated under these conditions, as evidenced by the generation of a 145-kDa fragment of α-spectrin, and that calpains are involved in apoptosis, because PD150606 induced a strong reduction in apoptosis (approximately 62%) but reduced caspase-3, caspase-7 and PARP activation by approximately 25%, which was significantly less than the effect observed upon TcdB treatment, without changing caspase-9 activation and while slightly reducing Bid expression (18%). Therefore, upon TcdB + CK treatment, calpains also activate apoptotic execution signalling in a mitochondrial-independent but caspase-3- and caspase-7-dependent manner. However, the role of calpains in effector caspase activation is minor, because after inhibition by PD150606, the inhibitory effect on apoptosis did not correlate with a similar reduction in activation of the effector caspase-3, caspase-7, and PARP, which suggests that the effectors described above can also be cleaved by cathepsin B. Therefore, upon treatment with TcdB and TcdB + CKs, PD150606 inhibited effector caspase activation due to calpain activation but could not inhibit the activation of cathepsin B, which contributes to apoptosis in a caspase-independent manner.

The increase in intracellular Ca^2+^ responsible of calpain activation does not seem to be due to the activation of L-type or T-type Ca^2+^ channels, because the specific inhibitors nifedipine and NiCl_2_ did not reduce apoptosis.

Our results suggest that three apoptotic pathways were activated upon treatment with both TcdB and TcdB + CKs: one due to the activation of cathepsin B, which was probably activated to a greater degree upon TcdB + CK treatment due to the synergistic effects of TNF-α and IFN-γ and the other two due to the activation of caspases and calpains, inducing predominantly caspase-dependent apoptotic signalling upon TcdB treatment and caspase-independent apoptotic signalling upon TcdB + CK treatment.

However, the first pro-apoptotic event induced by both TcdB and TcdB + CKs may be a rapid change in intracellular Ca^2+^ levels that triggers the apoptotic signalling pathways. It has been shown that Ca^2+^ is essential for the first events that occur when TcdB and cells interact [24, 66, 67] and act with TNF-α to help amplify the initial effect of TNF-α on the cell [30–33, 36–40]. After binding its receptor, TNF-α can activate the apoptotic pathway by activating caspase-8, which directly [30–33] or indirectly [36–40, 42–46] (through Bid cleavage and subsequent caspase-9 activation) activates the effectors caspase-3 and caspase-7. The results obtained with the specific caspase-8 inhibitor Z-IETD-fmk demonstrated that upon both TcdB and TcdB + CK treatment, apoptosis was significantly inhibited (approximately 28%). However, while the involvement of caspase-8 in the effects of TcdB + CKs was expected, the activation of caspase-8 upon TcdB treatment remains to be explained. Our data demonstrating caspase-8 activation are in agreement with the demonstration that caspase-8 was activated when apoptosis was induced by TcdA and TcdB in different cell types [17, 20, 21, 24], and our demonstration that EGC apoptosis was reduced by a caspase-8 inhibitor under both conditions is in agreement with the finding that caspase-8 activation was inhibited when the TcdA-induced apoptosis of T84 cells was inhibited with caspase-8 inhibitors [17] or glutamine and alanyl-glutamine [20]. However, our data differ with data obtained in other cell models of Tcd-induced apoptosis showing that caspase-8 inhibitors did not affect apoptosis induced by TcdB in Hep-2 cells and by TcdA in OVCAR3 cells, suggesting that in some experimental models, caspase-8 plays an ancillary role [21, 24], and confirm that the involvement of different caspases in Tcd-induced apoptosis depends on several factors, such as cell type, Tcd concentration and the patterns/levels of pro-apoptotic molecule activation.

An analysis of caspase-8 activation showed that Z-IETD-fmk in TcdB-induced EGC apoptosis did not affect the cleavage of caspase-3 or Bid expression but increased caspase-9 activation and inhibited the cleavage of caspase-7 and PARP. In contrast, in TcdB + CK-induced EGC apoptosis, Z-IETD-fmk inhibited caspase-3, caspase-9 and PARP cleavage and reduced Bid expression without affecting caspase-7 activation. Altogether, these results suggest that in apoptosis induced by TcdB + CKs, caspase-8 activation contributes to the transduction of pro-apoptotic signalling pathways by increasing Bid expression, leading to partial caspase-9 activation, which can contribute to caspase-3 activation and consequently to PARP activation. This indicates the partial involvement of mitochondrial apoptotic pathways. However, in apoptosis induced by TcdB, the reduction in apoptosis induced by Z-IETD-fmk was not accompanied by a significant reduction in caspase-3, Bid, caspase-9 or PARP signalling, indicating that caspase-8 contributes to apoptosis by directly cleaving caspase-7, and as we previously demonstrated that TcdB-induced apoptosis does not involve the mitochondrial apoptotic pathway [9].

Although caspase-independent Bid cleavage was found to be involved in apoptosis signalling in TcdA-induced T84 cell apoptosis [20], our results did not show the cleavage of Bid (a marker of extrinsic or TNF-α-associated apoptosis signalling) upon treatment with TcdB or TcdB + CKs, and only a slight increase in Bid expression was observed upon TcdB + CK treatment, which seemed to be induced by the CKs alone rather than by TcdB + CKs likely through regulation of cathepsin B activation. Our demonstration that Bid was not cleaved in TcdB- and TcdB + CKs-induced EGC apoptosis is in agreement with the demonstration that no significant increase in Bid cleavage was observed when apoptosis was induced by TcdA in non-transformed human colonocytes [18], and rather an increased in Bid expression was observed when apoptosis was induced by TcdB in human endothelial cells [26], confirming that the activation of apoptotic signalling strongly depends on the cell model, concentration of Tcds and patterns/levels of pro-apoptotic molecule activation.

The observation that pan-caspase inhibitor Q-VD-OPh (10-50 μM) and high doses of Z-DEVD-fmk strongly increase the cleavage of executioner caspase-3 under both conditions add support to the fact that if we inhibited the caspases, the result is that the cathepsin B no longer controls caspase-3 and caspase-7 cleavage due to the activity of the calpains. It is therefore possible that are caspase-3 and caspase-7 that maintain the activity of cathepsin B that regulates their activation levels and once that they are inhibited or by pan-caspase inhibitors (BAF, Q-VD-OPh) or effector caspase inhibitor (Z-DEVD-fmk), in a model perturbed by TcdB gives this result. However, to these results could also contribute the complex interplay on regulation of protease activation because calpains and cathepsin B are known to cleave many important regulators of apoptosis as XIAP. Moreover, the observation that inhibition of caspases with pan-caspase inhibitor (e.g. BAF) leads to an increase in the α-spectrin cleavage into the 145-kDa fragment (specific of calpain activity) indicates that also calpain activation is itself regulated by caspases.

We have also attempted to elucidate this complex interplay of apoptotic pathways through the association of different inhibitors. The combination of Z-DEVD-fmk with PD150606 and Z-DEVD-fmk with CA-074Me, to sum up BAF or Q-VD-OPh effects in apoptosis showed that the combination of two inhibitors does not have a synergistic effect, but rather the same effect of the single inhibitor. This can be explained by the complex interplay of regulation of proteases involved where the inhibition of two apoptotic pathways leaves free the third apoptotic pathway that remains responsible of apoptosis not susceptible to inhibition, as further suggested by the following results reported in our study: (a) when we inhibit the caspases with pan caspase inhibitor (BAF, Q-VD-OPh but also with higher doses of Z-DEVD-fmk) the cleavage of effector caspases was not abolished but increased suggesting that this cleavage is likely due to upstream activation of calpains and cathepsin B that can be more activated when caspases were inhibited, (b) when we inhibit the caspases with pan-caspase inhibitor BAF the cleavage of α-spectrin into the 145-kDa product, specific of calpain activity, increases suggesting that calpains are strongly activated when caspases are inhibited and therefore the cleavage of α-spectrin increased. Then, calpains are more strongly activated when caspases are inhibited leading so to stronger caspase-3 and caspase-7 cleavage, (c) when we inhibit the cathepsin B with Ca-074Me the cleavage of effector caspases was strongly increased, suggesting that this cleavage is likely due to caspases and calpains that can be strongly activated when cathepsin B was inhibited.

Overall, our data indicate that induction and distal execution apoptotic signalling are mediated by several families of proteases (Fig. 12). Regarding induction of apoptotic signalling we have demonstrated that caspase-8 contributes to apoptosis cleaving caspase-3 and caspase-7 contributing to 30% apoptosis and in TcdB + CKs there was also a contribution of caspase-9 activation (Fig. 12). Further, we demonstrated that also calpains and cathepsin B contribute to the executioner caspase-3 and caspase-7 cleavage and calpains to more than 60% apoptosis while cathepsins to 35% apoptosis (Fig. 12). This can be also potentiated and differentially regulated in TcdB + CKs by the contemporaneity of activation of cytokine-self dependent signalling and by the mutual regulation of families of proteases between themselves contributing differently to apoptotic pathways in TcdB and TcdB + CKs (Fig. 12). Regarding execution apoptotic signalling, the limited inhibition of apoptosis by Z-DEVD-fmk suggests that there are apoptotic caspase-3 and caspase-7-independent pathways in which caspase-8, calpains, or cathepsin B could directly cleave PARP or other effector substrates and thus bypass the blockade of inhibited caspase-3 and caspase-7 (Fig. 12). Further, although our data exclude a role of tBid because we do not find its cleavage, we observed an increase in the expression of Bid that could alter the equilibrium between pro- and anti-apoptotic Bcl-2 family members in favour of the pro-apoptotic members above all in TcdB + CKs allowing the triggering of a mitochondria-dependent but caspase-independent execution of apoptosis.

When we also extended the period of intoxication up to 72 h, the results are very suggestive because they evidenced that the apoptosis process was dynamic. Very importantly, in cells treated with TcdB + CKs the apoptosis doubled at 48 h (48.8%) and more strongly increased at 72 h (60.6%), suggesting that in such situations the damage of the TcdB + CKs is further amplified through time, unlike what happens for TcdB alone. In cells treated with TcdB alone, apoptosis increased only at 72 h of 65% then the cells treated with TcdB alone go in apoptosis mainly at 24 h, and there is only a weak delayed apoptosis. With regard to TcdB, using the pan-caspase (BAF, Q-VD-OPh), calpain and cathepsin B inhibitors, the percentage of apoptotic cells present at 24 h remains approximately constant at 48 h and slowly increased at 72 h remaining anyway strongly inhibited at all times examined. This confirms that already at 24 h, the majority of cells susceptible to the apoptotic action of TcdB evidenced the apoptotic features so there are no cell death events that the inhibitor could have delayed in the first 24 h to manifest later. In the case of TcdB + CKs, on the other hand, without the inhibitor the apoptosis strongly increased progressively up to 72 h, highlighting a progressive increase in apoptosis also in cells, which after 24 h are not yet apoptotic. However, even in this case in presence of the pan-caspase, calpain and cathepsin B inhibitors the percentage of apoptotic cells present at 24 h significantly increased at 48 h with all inhibitors and doubled at 72 h remaining anyway strongly inhibited at all time examined, indicating that the inhibitor is able to block apoptosis only in the cells programmed for apoptosis at 24 h but not for a portion of cells that are programmed to go into apoptosis at longer timepoints.

Apoptotic signalling is also strongly regulated by kinases such as JNK and AKT, which can exert opposing effects on processes such as cell death and survival and are involved in altering the balance between the pro-apoptotic and pro-survival arms of the TNF-α signalling cascade [27, 32, 33, 48–53, 63].

Regarding the role of JNK signalling in apoptosis induced by TcdB and TcdB + CKs, we found that TcdB under these conditions was not able to increase JNK phosphorylation in EGCs, but JNK inhibitor increased apoptosis under both conditions, with the effect greater upon treatment with TcdB; this finding is in line with findings of other models [27, 48, 49, 53], and in our model, JNK was found to contribute to the survival of EGCs in both TcdB- and TcdB + CK-induced apoptosis, but the mechanism requires further in-depth studies.

Because AKT plays a central role in regulating both cell death and survival signals, we investigated the possible role of AKT in our model [50–52, 63]. An important but problematic aspect highlighted by our results is that level of the phosphorylated form of AKT increased upon the induction of apoptosis by TcdB and even more so when apoptosis was induced by TcdB + CKs. Therefore, we wondered whether phosphorylated AKT exerts a pro-apoptotic effect or whether its levels increased in an attempt by the cell to counteract apoptosis. The inhibition of AKT phosphorylation by the specific inhibitor perifosine inhibited apoptosis after treatment with TcdB alone, indicating that phosphorylated AKT exerts a pro-apoptotic effect. However, likely due to the complex interaction between different pro-apoptotic signals activated by the synergistic effects of TcdB and CKs, we did not find a reduction in apoptosis with perifosine upon TcdB + CK treatment, suggesting that under these conditions, perifosine inhibits AKT activation, but inhibition of the pro-apoptotic activity of AKT does not overcome pro-apoptotic signalling activation by caspases, calpains, and cathepsins. Then, we aimed to clarify whether AKT would be activated by caspase, calpain, and cathepsin activation and the role of activated AKT in apoptosis induced by TcdB and TcdB + CKs using caspase, calpain, and cathepsin inhibitors. Overall, the results obtained in our model in agree with the results from other models [50–52, 63] suggest that AKT phosphorylation tends to play a protective role, based on an increase in its phosphorylated form, to antagonize the induction of apoptosis by TcdB and TcdB + CKs. Furthermore, these results suggest that that neither calpains nor caspases contribute to AKT phosphorylation, as their inhibition increased or did not affect the level of AKT phosphorylation under both conditions by either affecting apoptosis or altering caspase activation levels. In contrast, because the cathepsin B inhibitor reduced AKT phosphorylation, it is possible that cathepsins are involved in AKT phosphorylation. According to the results, when AKT phosphorylation was inhibited with perifosine, we found an increase in caspase-3 activation, suggesting an inverse correlation between caspase-3 activation and AKT phosphorylation. Furthermore, perifosine increased caspase activation under both conditions, and we found that apoptosis was reduced after TcdB treatment but unaffected by TcdB + CK treatment, suggesting that AKT activation promoted TcdB-induced apoptosis, but in TcdB + CK-induced apoptosis exerted an anti-apoptotic/pro-survival effect likely induced by TNF signalling. Our results on the effects of caspase, calpain, and cathepsin inhibitors indicated that AKT phosphorylation is caspase activation- and calpain activation-independent but cathepsin B activation-dependent. Moreover, our results indicated an inverse correlation between the caspase and AKT signalling pathways. In fact, AKT phosphorylation increased when caspases were inhibited, as observed with caspase and calpain inhibitors, but decreased when caspases were activated, as observed with a cathepsin B inhibitor. These results are consistent with the observation that perifosine decreased AKT phosphorylation but increased caspase activation. However, further studies are necessary to define the role in apoptosis because the use of perifosine suggested that increased AKT phosphorylation, had a pro-apoptotic effect, particularly upon treatment with TcdB alone, while the use of caspase, calpain, and cathepsin inhibitors suggested that had an anti-apoptotic effect. These apparently contradictory results on the role of AKT activation suggested by the use of perifosine or caspase, calpain, and cathepsin inhibitors can be explained by the complexity and interplay between apoptosis signalling activated by TcdB and TcdB + CKs and by the observation that AKT activation occurs before or concurrent with the activation of apoptotic signalling and is not dependent on caspase or calpain activation.

The activation of NF-κB by TNF-α is important because the translocation of NF-κB to the nucleus after degradation of its inhibitor IκBα induces the synthesis of various anti-apoptotic molecules [32, 33, 47–51]. TcdB induced an increase in IκBα suggesting the inhibition of NF-κB, while TcdB + CKs reduced the level to the control value, suggesting the reactivation of NF-κB signalling.

This suggests that TNF-α attempted to activate an anti-apoptotic response, but the ultimate effect of stimulation was pro-apoptotic.

In conclusion, TcdB induced apoptosis in EGCs due to the activation of three signalling pathways (Fig. 12), two of which were activated by calpains and caspases in a caspase-dependent and -independent manner and one of which was activated by cathepsin B in a caspase-3- and caspase-7-independent manner (Fig. 12). The activation of cathepsin B was probably due to inhibition of Rac1, which, by altering the cytoskeleton, can modify the permeability of the lysosomal membrane, favouring the release of cathepsin B in the cytoplasm, which activates caspase-independent apoptotic signalling. Upon TcdB + CK treatment, apoptotic signalling due to TcdB was enriched and accompanied by TNF-α activity which helped to enhance apoptosis induced by TcdB, which, by blocking Rac1, prevents TNF-α from partially activating anti-apoptotic signalling. Furthermore, it is possible that the earliest key event in TcdB activity is an increase in intracellular Ca^2+^ levels with consequent activation of calpains, an event that can be further enhanced by the CKs themselves. However, the involvement of NF-κB, AKT, JNK and ROS in cytotoxic synergism between TcdB and CKs requires deeper investigation.

From this study, some important, basic implications and other insights concerning the pathogenesis of *C. difficile* emerge:

(a) The role of cathepsin B in cleaving caspase-3, caspase-7 and PARP to generate activated fragments is an important aspect of the complex reciprocal regulatory network between the various protease families. However, it remains to be understood how high levels of caspase-3 and caspase-7 cleavage induced by cathepsin B inhibition do not promote apoptosis.

(b) The ability of TcdB to activate at least three apoptotic signalling pathways, which was also enhanced by CKs, increased the ability of *C. difficile* to induce progressive cell death in the various types of immune cells recruited to the site of infection, which may have intrinsic resistance to a single apoptotic signalling.

(c) Enhancement of the cytotoxic activity of TcdB by two pro-inflammatory CKs may have important consequences in vivo, where CDI is characterized by a strong inflammatory response. Furthermore, Tcds themselves can induce the production of TNF-α by macrophages, potentially generating an autocrine loop that enhances cytotoxicity in the presence of TcdB. However, whether other pro-inflammatory CKs, such as IL-1β and IL-6, can enhance the cytotoxic activity of TcdB remains to be verified.

While the inflammatory response of the host to various infections contribute to contain the infection, on the other hand, pathogens have evolved the ability to divert the inflammatory response to enhance the infection and to survive longer in the host; thus, the inflammatory response can be an important therapeutic target.

## Supplementary Information

Below is the link to the electronic supplementary material.

Supplementary file1 (DOCX 1616 kb)

## Data Availability

All materials are available on request, and the manuscript includes supplemental information submitted electronically.

## Abbreviations

*C. difficile*: *Clostridioides difficile*
CDI: *Clostridioides difficile* Infection
TcdA: *Clostridioides difficile* Toxin A
TcdB: *Clostridioides difficile* Toxin B
EGCs: Enteric glial cells
PARP: Poly(adenosine diphosphate-ribose) polymerase
Bcl-2: B-cell lymphoma 2
IL-1β: Interleukin-1 beta
TNF-α: Tumour necrosis factor alpha
IFN-γ: Interferon gamma
IL-6: Interleukin 6
CKs: TNF-α plus IFN-γ
Ca^2+^: Calcium
Bid: BH3-interacting domain death agonist
Tcds: *Clostridioides difficile* Toxins
ALLM: N-Acetyl-Leu-Leu-methioninal
Bax: Bcl-2-associated X protein
JNK: C-Jun N-terminal kinase
ROS: Reactive oxygen species
NF-κB: Nuclear factor-kappa B
PI3K: Phosphatidylinositol 3-kinase
BAF: Boc-Asp(OMe)-fluoromethyl ketone
Q-VD-OPh: Q-Val-Asp-Oph
Z-DEVD-fmk: Z-Asp-Glu-Val-Asp-fluoromethylketone
Z-IETD-fmk: Z-Ile-Glu(OMe)-Thr-Asp(OMe)-fluoromethylketone
PD150606: 3-(4-Iodophenyl)-2-mercapto-2-propenoic acid
CA-074Me: [L-3-*trans*-(Propylcarbamoyl)oxirane-2-carbonyl]-l-isoleucyl-l-proline methyl ester
CST: Cell signaling technology
NiCl_2_: Nickel(II) chloride
BAPTA-AM: 1,2-Bis(2-aminophenoxy)ethane-*N*,*N*,*N*′,*N*′-tetraacetic acid-acetoxymethyl ester
EGTA: Ethylene glycol-bis(β-aminoethyl ether)-*N*,*N*,*N*′,*N*′-tetraacetic acid
SDS-PAGE: Sodium dodecyl sulphate–polyacrylamide gel electrophoresis
ab: Antibody
pJNK: Phospho-JNK
pAKT: Phospho-AKT
IkBα: Nuclear factor of kappa light polypeptide gene enhancer in B-cell inhibitor alpha
mAb: Monoclonal Ab
HRP: Horseradish peroxidase
Bcl-X_L_: B-cell lymphoma X long isoform
