# Supplementary material for: Clostridioides difficile toxin B alone and with pro-inflammatory cytokines induces apoptosis in enteric glial cells by activating three different signalling pathways mediated by caspases, calpains and cathepsin B
Source: Cell Mol Life Sci. 2022 Jul 22;79(8):442. doi: 10.1007/s00018-022-04459-z (PMC9304068; doi:10.1007/s00018-022-04459-z)
Supplement: Supplementary file 1 — Supplementary file1 (DOCX 1616 kb) [file 18_2022_4459_MOESM1_ESM.docx]

***CLOSTRIDIOIDES DIFFICILE* TOXIN B ALONE AND WITH PRO-INFLAMMATORY CYTOKINES INDUCES APOPTOSIS IN ENTERIC GLIAL CELLS BY ACTIVATING THREE DIFFERENT SIGNALLING PATHWAYS MEDIATED BY CASPASES, CALPAINS AND CATHEPSIN B**

**CELLULAR AND MOLECULAR LIFE SCIENCES**

**Katia Fettucciari,^1*#^ Flavien Marguerie,^2^ Alessandro Fruganti,^3^ Andrea Marchegiani,^3^ Andrea Spaterna,^3^ Stefano Brancorsini,^4^ Pierfrancesco Marconi,^1^ Gabrio Bassotti^5,6#^**

*Corresponding author:

**Dr. Katia Fettucciari**

Department of Medicine and Surgery, University of Perugia, Medical School, - Edificio B - IV piano, Piazza Lucio Severi 1, -06132- Perugia- Italy

Phone: +39755858124

Email: [katia.fettucciari@unipg.it](mailto:katia.fettucciari@unipg.it)

**
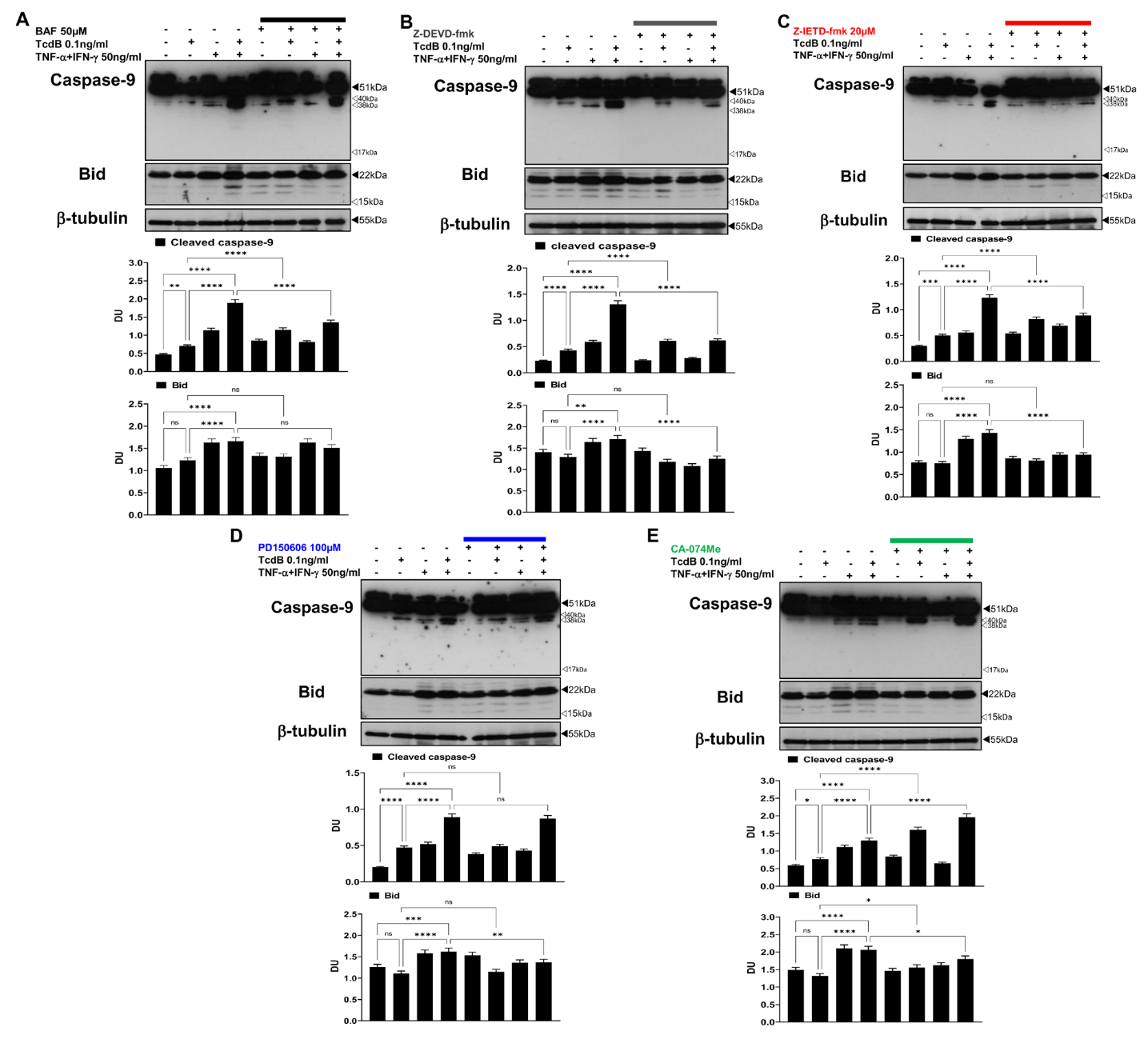
**

**Fig. S1 BAF, Z-DEVD-fmk, Z-IETD-fmk, PD150606 and CA-074Me had different effects on caspase-9 activation and BID expression.**

**A-E** Whole-cell lysates from EGCs that were or were not pre-treated for 1 h with BAF (50 µM) (**A**), Z-DEVD-fmk (2 µM) (**B**), Z-IETD-fmk (20 µM) (**C**), PD150606 (100 µM) (**D**), or CA-074Me (10 µM) (**E**), were or were not exposed to TcdB (0.1 ng/ml) for 1.5 h, and were or were not stimulated or not with TNF-α (50 ng/ml) plus IFN-γ (50 ng/ml) (CKs) were prepared at 24 h and subjected to SDS-PAGE. **A-E** Filters were probed with anti-caspase-9 Ab, stripped and re-probed with anti-Bid Ab, and finally stripped and re-probed with anti-β-tubulin Ab. Blots are representative of three independent experiments. Intact protein (solid arrow) and active fragment (open arrow) are indicated. The graphs represent the mean ± standard deviation of densitometric analysis of cleaved caspase-9 or Bid relative to β-tubulin in three different experiments. Statistical analysis was performed by one-way ANOVA and Tukey’s multiple comparisons test. *P <0.05, ** P < 0.01, *** P <0.001, ****P< 0.0001, ns P >0.05.


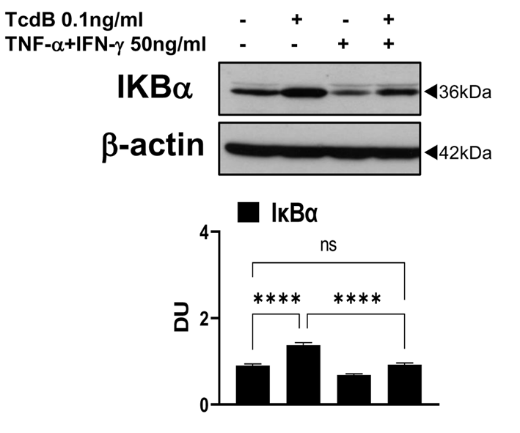


**Fig.** **S2** **IκBα expression was increased after TcdB treatment and decreased after TcdB+CK treatment.**

Whole-cell lysates from EGCs that were or were not exposed to TcdB (0.1 ng/ml) for 1.5 h, and were or were not stimulated with TNF-α (50 ng/ml) plus IFN-γ (50 ng/ml) were prepared at 24 h and subjected to SDS-PAGE. The filter was probed with anti-IκBα Ab and then stripped and re-probed with anti-β-actin Ab. Blots are representative of three independent experiments. The graph represents the mean ± standard deviation of densitometric analysis of IκBα relative to β-actin in three different experiments. Statistical analysis was performed by one-way ANOVA and Tukey’s multiple comparisons test. *P <0.05, ** P < 0.01, *** P <0.001, ****P< 0.0001, ns P >0.05.

**
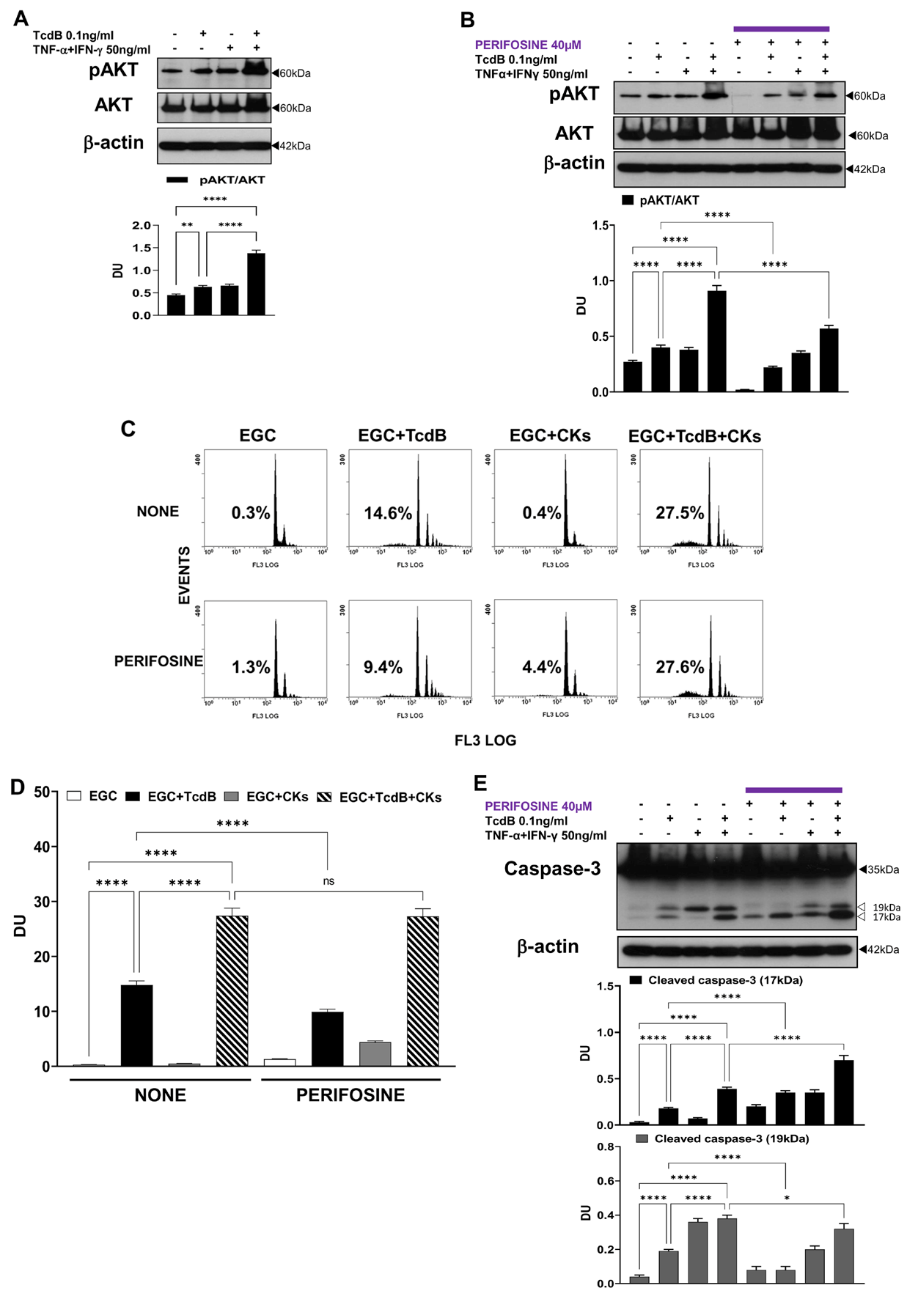
**

**Fig. S3** **Activation of AKT and effect of perifosine on AKT phosphorylation, apoptosis and caspase-3 activation in EGCs treated with TcdB and TcdB+CKs.**

**A** Whole-cell lysates from EGCs that were or were not exposed to TcdB (0.1 ng/ml) for 1.5 h, and were or were not stimulated with TNF-α (50 ng/ml) plus IFN-γ (50 ng/ml) (CKs) were prepared at 24 h and subjected to SDS-PAGE. The filter was probed with anti-pAKT Ab, then stripped and re-probed with anti-β-actin Ab, then stripped and reprobed with AKT total Ab. Blots are representative of three independent experiments. The graph represents the mean ± standard deviation of densitometric analysis of pAKT relative to AKT in three different experiments. **B-E** EGCs were or were not pre-treated for 1 h with perifosine (40 µM), were or were not exposed to TcdB (0.1 ng/ml) for 1.5 h and were or were not stimulated with TNF-α (50 ng/ml) plus IFN-γ (50 ng/ml) (CKs). Cells from all experimental conditions were recovered at 24 h to prepare whole-cell lysates for SDS-PAGE and Western blot analysis (**B, E**) and to evaluate apoptosis (**C, D**). **B** The filter was probed with anti-pAKT Ab and then stripped and re-probed with anti-β-actin Ab, then stripped and reprobed with AKT total Ab. Blots are representative of three independent experiments. The graph represents respective densitometric analysis of pAKT relative to AKT in three different experiments. **C, D** Apoptosis was evaluated by measuring the percentage of hypodiploid nuclei by flow cytometry. DNA fluorescence flow cytometric profiles with percentages of hypodiploid nuclei of one experiment representative of three (**C**) and graph showing the mean ± standard deviation of percentage of hypodiploid nuclei obtained in three different experiments (**D**) are shown. **E** The filter was probed with anti-caspase-3 Ab and then stripped and re-probed with anti-β-actin Ab. Blots are representative of three independent experiments. Intact protein (solid arrow) and active fragment (open arrow) are indicated. The graphs represent the mean ± standard deviation of densitometric analysis of cleaved caspase-3 (17kDa), or cleaved caspase-3 (19kDa), relative to β-actin in three different experiments. **A, B, D, E** Statistical analysis was performed by one-way ANOVA and Tukey’s multiple comparisons test. *P <0.05, ** P < 0.01, *** P <0.001, ****P< 0.0001, ns P >0.05.


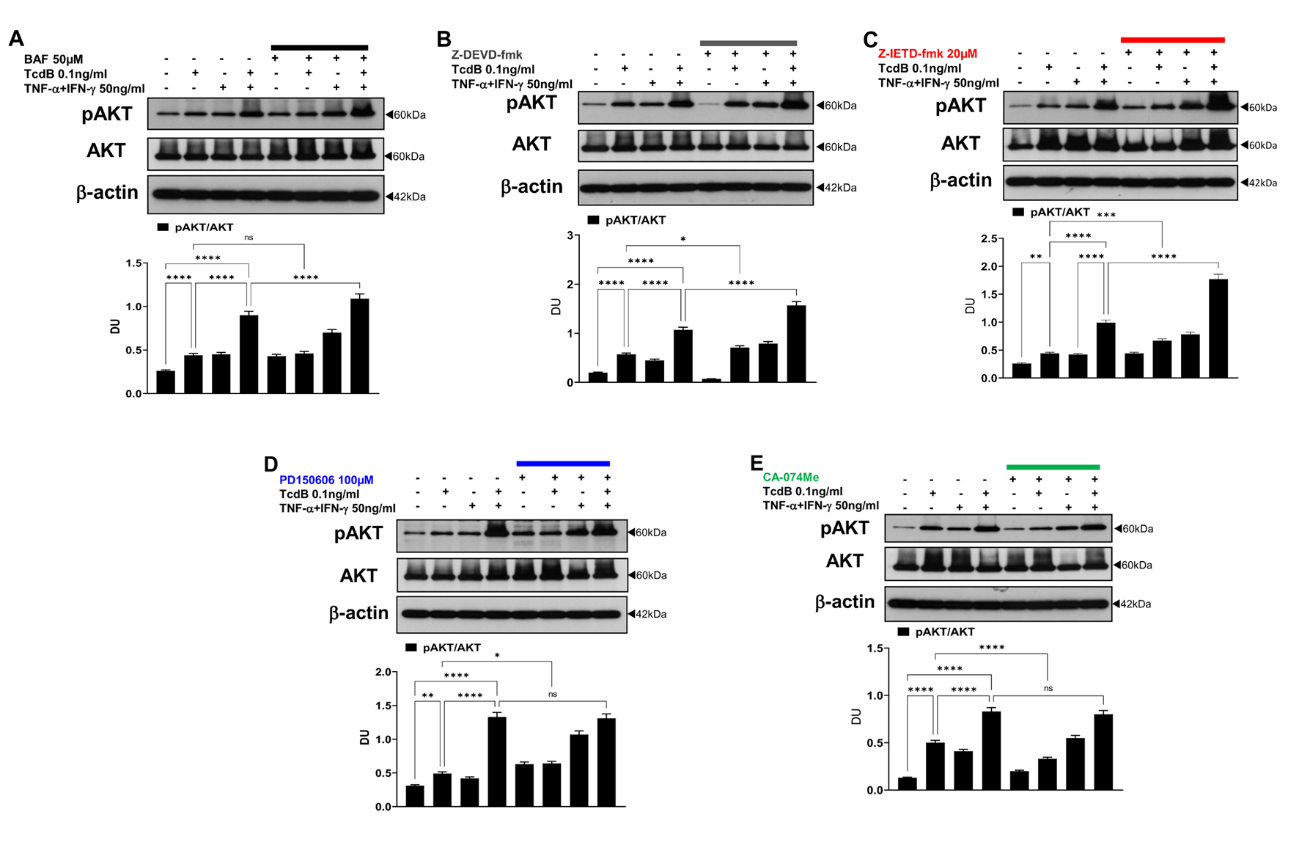


**Fig. S4 BAF, Z-DEVD-fmk, Z-IETD-fmk, and PD150606 increased AKT phosphorylation after TcdB and TcdB+CK treatment, while CA-074Me reduced AKT phosphorylation.**

**A-E** Whole-cell lysates from EGCs that were or were not pre-treated for 1 h with BAF (50 µM) (**A**), Z-DEVD-fmk (2 µM) (**B**), Z-IETD-fmk (20 µM) (**C**), PD150606 (100 µM) (**D**), or CA-074Me (10 µM) (**E**), were or were not exposed to TcdB (0.1 ng/ml) for 1.5 h, and were or were not stimulated with TNF-α (50 ng/ml) plus IFN-γ (50 ng/ml) (CKs) were prepared at 24 h and subjected to SDS-PAGE. **A-E** Filters were probed with pAKT Ab and then stripped and re-probed with anti-β-actin Ab, then stripped and reprobed with AKT total Ab. Blots are representative of three independent experiments. The graph represents respective densitometric analysis of pAKT relative to AKT in three different experiments. **A-E** Statistical analysis was performed by one-way ANOVA and Tukey’s multiple comparisons test. *P <0.05, ** P < 0.01, *** P <0.001, ****P< 0.0001, ns P >0.05.


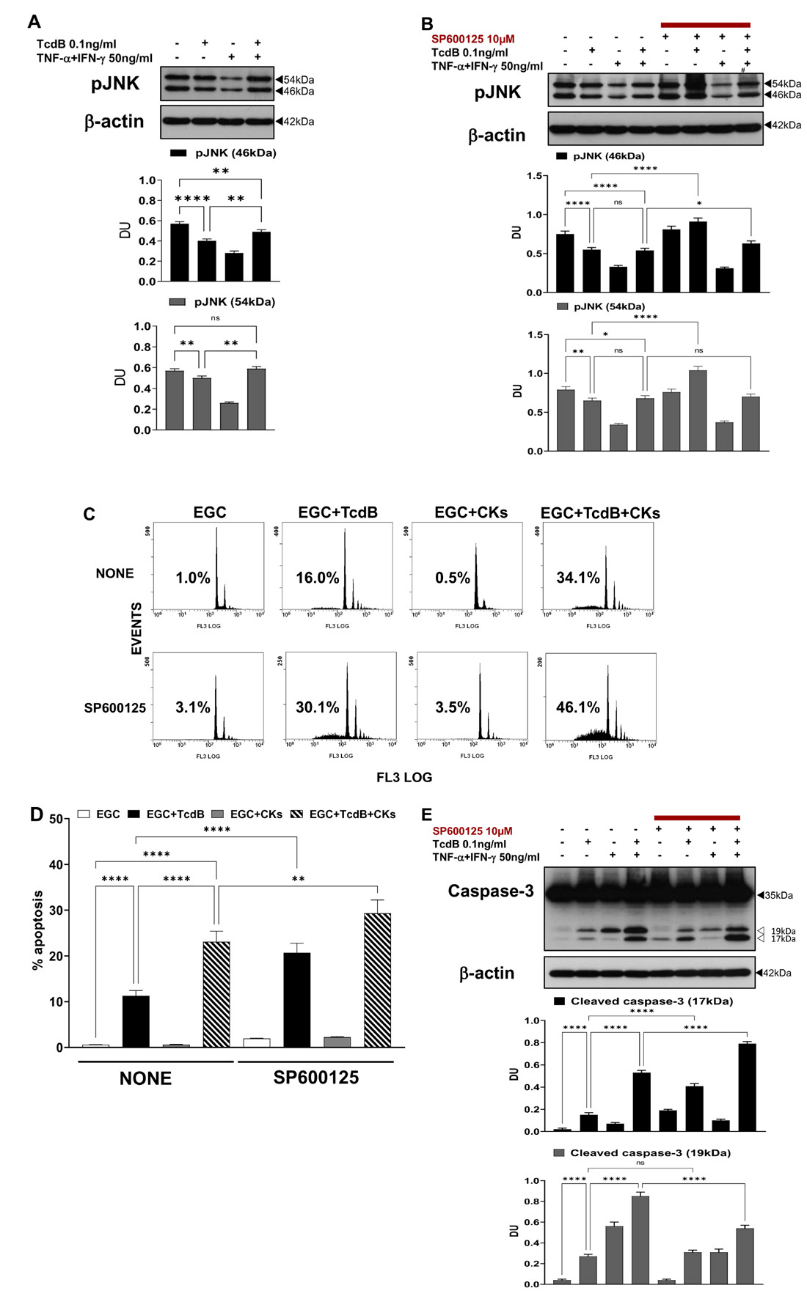


**Fig. S5 Activation of JNK and the effect of SP600125 on JNK phosphorylation, apoptosis and caspase-3 activation**

**in EGCs treated with TcdB and TcdB+CKs.**

**A** Whole-cell lysates from EGCs that were or were not exposed to TcdB (0.1 ng/ml) for 1.5 h and were or were not stimulated with TNF-α (50 ng/ml) plus IFN-γ (50 ng/ml) (CKs) were prepared at 24 h and subjected to SDS-PAGE. The filter was probed with anti-pJNK Ab and then stripped and re-probed with anti-β-actin Ab. Blots are representative of three independent experiments. The graphs represent the mean ± standard deviation of densitometric analysis of pJNK (46kDa), or pJNK (54kDa), relative to β-actin in three different experiments. **B-E** EGCs were or were not pre-treated for 1 h with SP600125 (10 µM), were or were not exposed to TcdB (0.1 ng/ml) for 1.5 h, and were or were not stimulated with TNF-α (50 ng/ml) plus IFN-γ (50 ng/ml) (CKs). Cells from all experimental conditions were recovered at 24 h to prepare whole-cell lysates for SDS-PAGE and Western blot analysis (**B, E**) and to evaluate apoptosis (**C, D**). **B** The filter was probed with anti-pJNK Ab and then stripped and re-probed with anti-β-actin Ab. Blots are representative of three independent experiments. The graphs represent the mean ± standard deviation of densitometric analysis of pJNK (46kDa), or pJNK (54kDa), relative to β-actin in three different experiments. **C, D** Apoptosis was evaluated by measuring the percentage of hypodiploid nuclei by flow cytometry. DNA fluorescence flow cytometric profiles with percentages of hypodiploid nuclei of one experiment, representative of five (**C**) and graph showing the mean ± standard deviation of percentage hypodiploid nuclei obtained in five different experiments (**D**) are shown. **E** The filter was probed with anti-caspase-3 Ab and then stripped and re-probed with anti-β-actin Ab. Blots are representative of three independent experiments. Intact protein (solid arrow) and active fragment (open arrow) are indicated. The graphs represent the mean ± standard deviation of densitometric analysis of cleaved caspase-3 (17kDa), or cleaved caspase-3 (19kDa), relative to β-actin in three different experiments. **A, B, D, E** Statistical analysis was performed by one-way ANOVA and Tukey’s multiple comparisons test. *P <0.05, ** P < 0.01, *** P <0.001, ****P< 0.0001, ns P >0.05.
